# Serum extracellular vesicular miR-21-5p is a predictor of the prognosis in idiopathic pulmonary fibrosis

**DOI:** 10.1186/s12931-016-0427-3

**Published:** 2016-09-05

**Authors:** Tomonori Makiguchi, Mitsuhiro Yamada, Yusuke Yoshioka, Hisatoshi Sugiura, Akira Koarai, Shigeki Chiba, Naoya Fujino, Yutaka Tojo, Chiharu Ota, Hiroshi Kubo, Seiichi Kobayashi, Masaru Yanai, Sanae Shimura, Takahiro Ochiya, Masakazu Ichinose

**Affiliations:** 1 Department of Respiratory Medicine, Tohoku University Graduate School of Medicine, 1-1 Seiryo-machi, Aoba-ku, Sendai, 980-8574 Japan; 2 Division of Molecular and Cellular Medicine, National Cancer Center Research Institute, 5-1-1 Tsukiji, Chuo-ku, Tokyo, 104-0045 Japan; 3 Department of Advanced Preventive Medicine for Infectious Disease and Regenerative Medicine, Tohoku University Graduate School of Medicine, 2-1 Seiryo-machi, Aoba-ku, Sendai, 980-8575 Japan; 4 Department of Respiratory Medicine, Japanese Red Cross Ishinomaki Hospital, 71 Nishimichishita, Hebita-aza, Ishinomaki, 986-8522 Japan; 5 Hikarigaoka Spellman Hospital, 6-7-1 Higashisendai, Miyagino-ku, Sendai, 983-0833 Japan

## Abstract

**Background:**

Idiopathic pulmonary fibrosis (IPF) is a disease with a poor prognosis. Although the median survival is 3 years, the clinical course varies to a large extent among IPF patients. To date, there has been no definitive prognostic marker. Extracellular vesicles (EVs) are known to hold nucleic acid, including microRNAs, and to regulate gene expression in the recipient cells. Moreover, EVs have been shown to express distinct surface proteins or enveloped microRNAs depending on the parent cell or pathological condition. We aimed to identify serum EV microRNAs that would be prognostic for IPF.

**Methods:**

To determine target microRNAs in IPF, we measured serum EV microRNA expression profiles using microRNA PCR arrays in a bleomycin mouse model and validated the microRNAs in additional mice using RT-PCR. Secondly, we enrolled 41 IPF patients and conducted a 30-month prospective cohort study. Expression of serum EV miR-21-5p was normalized by dividing by the EV amount. The relative amount of EVs was measured using the ExoScreen method. We calculated the correlations between baseline serum EV miR-21-5p expression and other clinical variables. Furthermore, we determined if serum EV miR-21-5p can predict mortality during 30 months using the Cox hazard model. According to the median level, we divided the IPF patients into two groups. Then we compared the survival rate during 30 months between the two groups using the Kaplan-Meier method.

**Results:**

Serum EV miR-21-5p was elevated in both the acute inflammatory phase (day 7) and the chronic fibrotic phase (day 28) in the mouse model. In the clinical setting, serum EV miR-21-5p was significantly higher in IPF patients than in healthy control subjects. The baseline serum EV miR-21-5p was correlated with the rate of decline in vital capacity over 6 months. Furthermore, serum EV miR-21-5p was independently associated with mortality during the following 30 months, even after adjustment for other variables. In the survival analysis, IPF patients whose baseline serum EV miR-21-5p was high had a significantly poorer prognosis over 30 months.

**Conclusions:**

Our results suggest that serum EV miR-21-5p has potential as a prognostic biomarker for IPF.

**Electronic supplementary material:**

The online version of this article (doi:10.1186/s12931-016-0427-3) contains supplementary material, which is available to authorized users.

## Background

Idiopathic pulmonary fibrosis (IPF) is a chronic and progressive lung disease for which no treatment is capable of providing a complete cure [1–3]. The median survival for IPF patients from the time of diagnosis is approximately 3 years [3]. Recently, new therapeutic targets for IPF have been identified, and some of the proposed therapies are expected to slow its progression [2]. IPF patients differ in terms of the disease progression rate and prognosis, complicating the prediction of survival. The identification of prognostic predictors for IPF is important for determining who requires the most intensive therapies.

MicroRNAs are 22-nucleotide-long non-coding RNAs that function in the translational repression or degradation of target mRNA [4]. MicroRNAs have been shown to affect physiological and pathological conditions, including lung disease [5]. A recent investigation of fibrotic lung diseases showed that the expression levels of several microRNAs were significantly altered in fibrotic lungs, suggesting that microRNAs contribute to the development and progression of fibrotic lung diseases [5]. Therefore, microRNAs have received considerable attention as potential therapeutic targets in IPF, as well.

Exosomes are one of the major components of extracellular vesicles (EVs) [6]. Recently, EVs, including exosomes, have been considered as novel tools for intercellular communication because EVs contain various proteins and nucleic acids including microRNAs [7]. MicroRNAs in EVs can be transferred to target cells to regulate gene expression and cell function [8–10]. EVs and enveloped microRNAs have been shown to function in physiological and pathological conditions [11–15]. EVs and enveloped microRNAs within biological fluids (e.g., circulating blood) have also attracted attention as novel biomarkers of diseases such as cancer because the components and secretion dynamics of EVs vary according to their cellular origin and environment [16, 17].

In this study, we explored the possibility that microRNAs of serum EVs changed during lung fibrosis and could serve as prognostic biomarkers of IPF. We examined the levels of serum EV microRNAs in a mouse model of lung fibrosis via quantitative PCR array, which revealed that miR-21-5p was significantly increased in serum EVs of the mouse model. Accordingly, we evaluated the levels of miR-21-5p in serum EVs (serum EV miR-21-5p) after adjusting for difference in the quantity of serum EVs among IPF patients.

## Methods

### Reagents

Total Exosome Isolation reagent (Thermo Fisher Scientific, Waltham, MA, USA) was used for EV purification from serum. The miRNeasy Mini Kit and Syn-cel-miR-39-3p miScript miRNA Mimic for serum EV RNA purification was purchased from QIAGEN (Hilden, Germany). The following antibodies were used for ExoScreen detection of EVs; biotin-conjugated anti-mouse CD9 (clone MZ3, Biolegend, San Diego, CA, USA), anti-mouse CD9 (clone MZ3, Biolegend), and anti-human CD9 (clone 12A12, Shionogi & Co., LTD, Osaka, Japan). ChromaLink™ Biotin Labelling Kit (Solulink, Inc., San Diego, CA, USA) was used for biotinylation of anti-human CD9 antibody. AlphaLISA reagents (PerkinElmer, Waltham, MA, USA) for ExoScreen included AlphaScreen Streptavidin Donor Beads, unconjugated AlphaLISA Acceptor Beads and AlphaLISA Universal Buffer. Conjugation of AlphaLISA Acceptor Beads to anti-human or -mouse CD9 antibodies was performed according to the manufacturer’s protocol.

### Cell cultures

A mouse lung cancer cell line (Lewis lung carcinoma (LLC)) and a mouse mesenchymal cell line (KUM-10) were obtained from RIKEN Cell Bank, Ibaraki, Japan. LLC and KUM-10 were cultured in DMEM with 10 % heat-inactivated fetal bovine serum (FBS) and an antibiotic-antimycotic solution (Thermo Fisher Scientific) at 37 °C in 5 % CO_2_.

### Preparation of conditioned media and EVs in media

The cells were washed with phosphate-buffered saline (PBS), and the culture medium was replaced with advanced Dulbecco’s Modified Eagle Medium for LLC and KUM-10 cells. After incubation for 48 h, the conditioned media were collected and centrifuged at 2000 g for 10 min at 4 °C. To thoroughly remove cellular debris, the supernatant was filtered through a 0.22 μm filter (EMD Millipore, Billerica, MA, USA). To prepare EVs, the conditioned media were ultracentrifuged at 110,000 g for 70 min at 4 °C. The pellets were washed with 11 mL of PBS, ultracentrifuged at 110,000 g for 70 min at 4 °C and resuspended in PBS. The protein content of the putative EV fraction was measured using a BCA protein assay (Thermo Fisher Scientific).

### ExoScreen assay

The detailed principle and analytical methods were presented in a previous report [18]. Briefly, a 96-well half-area plate was filled with 5 μL of sample, 5 nM biotinylated antibodies and 50 μg/mL AlphaLISA acceptor bead-conjugated antibodies against mouse or human CD9 in the universal buffer. The volume of each reagent was 10 μL. The plate was then incubated for 3 h at room temperature. Without a washing step, 25 μL of 80 μg/mL AlphaScreen streptavidin-coated donor beads were added. The reaction mixture was incubated in the dark for another 30 min at room temperature, and the plate was then read on a PHERAstar FS microplate reader (BMG LABTECH, Ortenberg, Germany) using the AlphaLISA mode (excitation wavelength of 680 nm and emission detection set to 615 nm). Background signals obtained from PBS were subtracted from the measured signals.

### The animal model of pulmonary fibrosis

All animal experiments were approved by the Tohoku University Animal Experiment Ethics Committee and performed in accordance with the Regulations for Animal Experiments and Related Activities at Tohoku University.

Seven- to 8-week-old male C57BL/6 mice were used in our experiments. C57BL/6 mice were purchased from CLEA Japan (Yokohama, Japan). All mice were housed in a specific pathogen-free facility and maintained under constant temperature (24 °C), humidity (40 %), and light cycle (8:00 A.M. to 8:00 P.M.), with food and water provided ad libitum. To induce pulmonary fibrosis, mice were treated intratracheally with bleomycin hydrochloride (Nippon Kayaku, Tokyo, Japan) on day 0 as described in our previous study [19]. Briefly, mice were anesthetized with ketamine via intraperitoneal injection and were then injected with 0.04 mg of bleomycin hydrochloride in 100 μl of saline through a 27G needle inserted between the cartilaginous rings of the trachea. Circulating blood was harvested 7, 14 or 28 days after instillation. Collected blood was incubated at room temperature for 1 h and centrifuged for 15 min at 1500 g at 4 °C. The serum was transferred to a new tube and centrifuged again for 30 min at 2500 g at 4 °C to remove cells and debris. The clarified serum was transferred for further examination.

### Subjects and specimens

This study was approved by the ethics committees at Tohoku University School of Medicine, Japanese Red Cross Ishinomaki Hospital and Hikarigaoka Spellman Hospital. All subjects gave written informed consent. This study is registered with UMIN-CTR, number UMIN000017403.

Human peripheral blood was obtained from patients or healthy volunteers at Japanese Red Cross Ishinomaki Hospital (Ishinomaki, Japan) or at Hikarigaoka Spellman Hospital (Sendai, Japan). Serum was obtained by centrifuging these specimens, aliquoted, and stored at −80 °C until used in analyses of serum EV. Forty-one patients with idiopathic pulmonary fibrosis (IPF) and 21 healthy controls were included (Table 3). Diagnoses were made according to the American Thoracic Society (ATS)/European Respiratory Society (ERS) statement based on clinical evaluation, high-resolution computed tomography, histology, and laboratory findings [20]. Emphysematous lesions were detected by CT and evaluated with Goddard LAA score [21]. The patients whose Goddard LAA score is ≥1 were considered as having emphysema. The expert clinicians who analysed the information were blinded to the diagnoses associated with the experimental laboratory tests. Usual interstitial pneumonia was confirmed by surgical biopsy in 15 of the IPF patients (Table 3). The patients were recruited at the time of diagnosis. The collection of blood samples and pulmonary function tests were performed at entry. IPF patients were followed for 30 months except in cases of death or failure to visit the hospital. Survival status was obtained from visits to the hospitals and telephone interviews. During the follow-up periods, the patients received pulmonary function evaluations 6 months after enrolment.

### Preparation of EVs from mouse or human serum samples

Mouse or human serum samples were centrifuged at 10,000 g for 10 min to remove cells and debris. Subsequently, we extracted EVs from serum using a commercial extracting reagent (Total Exosome Isolation from serum, Thermo Fisher Scientific) as previously described [22]. Briefly, we mixed 250 μL of centrifuged serum with 50 μL of an extracting reagent. The samples were incubated at 4 °C for 30 min and then centrifuged at room temperature at 10,000 g for 10 min. The supernatant was discarded and the EV pellet was resuspended in 100 μL of PBS. The EV suspension was used for further examination.

### Extraction of serum EV RNA and synthesis of cDNA

Total RNA was extracted from serum EV using a miRNeasy Mini Kit (QIAGEN, Hilden, Germany). Synthetic *C. elegans* miR-39 (QIAGEN) was added to samples of the serum EV suspension to control for variations during the preparation of total RNA. The miScript II RT Kit was used for reverse transcription of microRNAs into cDNA.

### MicroRNA PCR array in mouse serum EVs

The Mouse Serum miScript miRNA PCR Array (QIAGEN, MIMM-106Z) was used according to the manufacturer’s instructions for the comprehensive analyses of the expression levels of the microRNAs in the serum EV in a mouse model of pulmonary fibrosis. Briefly, template cDNA that had been synthesized from the mouse serum EV RNA was added to each well of the miScript miRNA PCR array plate. The plate was placed on the real-time cycler (StepOne Plus, Thermo Fisher Scientific) and run. The data were analysed using the web-based miScript miRNA PCR Array data analysis tool (QIAGEN). MicroRNAs were considered not detectable when Ct > 35. We also excluded the microRNAs for which the Ct > 30 in either group for further analyses on the basis that this indicated that the expression level was relatively low, which could cause greater variations in the fold-change results. The delta Ct value (target microRNA Ct–cel-miR-39 Ct) in each sample was calculated. The average of the delta Ct values in each sample group was then calculated and used to analyse the fold-changes of serum EV microRNAs compared with non-treated controls. The expression levels of microRNAs were normalized by dividing them by the amount of EVs in each serum EV sample, as determined by the ExoScreen assay. To control the false discovery rate for multiple comparisons, the Benjamini-Hochberg procedure was used [23]. The *q*-value for the fold-change for selecting the candidate microRNAs was less than 0.05.

### Quantification of microRNAs in serum EVs

The microRNAs were quantified by real-time PCR using the miScript Primer Assay (QIAGEN). The real-time RT-PCR detection of the *C. elegans* miR-39 was also performed for the normalization of the real-time RT-PCR results of the endogenous microRNAs in the sample. This procedure corrects for variations during the RNA preparation, cDNA synthesis, and real-time PCR. To determine the copy numbers of human miR-21-5p in the serum samples, standard curves were prepared using serial dilutions of synthetic human miR-21-5p (Bioneer, Daejeon, Korea). The relative amount of the spiked cel-miR-39 in each sample was calculated using the serially diluted standard samples. This value was used for normalization between samples. The formula for this calculation is as follows: (The normalized amount of miR-21-5p or miR-155-5p in sample X) = (the pre-normalized amount of the microRNA in sample X) × (the amount of cel-miR-39 in the reference sample/the amount of cel-miR-39 in sample X). The reference sample was one of the non-treated control samples for mouse study or one of the IPF patient samples (the sample of IPF patient #1) for human study, respectively. The expression levels of the microRNAs were normalized by dividing them by the amount of EVs in each serum EV sample, as determined by the ExoScreen.

### Statistical analysis

The statistical analysis was performed using JMP Pro 11.0 (SAS Institute Inc., Cary, NC). In the animal experiments, the data are presented as the means ± SEM unless otherwise indicated. The Kruskal-Wallis test was used for multiple comparisons, and *p* < 0.05 was taken to represent statistical significance. We used the Benjamini-Hochberg procedure [23] to control the false discovery rate for multiple comparisons for the microRNA PCR array analyses. The *q*-value for the fold-change for selecting the candidate microRNAs was less than 0.05. In the clinical setting, the data are presented as the medians (IQR). The differences in the distribution of the categorical data between two groups were analysed using the Fisher exact test. The differences in continuous data between two groups were analysed using the Mann-Whitney *U* test. Correlations between miR-21-5p and other clinical variables were calculated using the Spearman rank correlation. In the correlation analysis of the baseline characteristics, the Cox proportional hazards model was used to determine the effect of various factors on mortality in IPF patients. These results are expressed as hazard ratios for death among those who had a factor of interest compared with those who did not have the factor. We calculated the median values for the normalized or non-normalized serum EV miR-21-5p levels in the 41 IPF patients. These values were determined to be 2.1 copies/SI or 1.25 × 10^7^ copies/mL, respectively. We divided the 41 IPF patients into the following two groups: those above and those below the median value. We also divided the IPF patients into the top-quartile versus the remaining subjects for the following analysis. Survival was evaluated using the Kaplan-Meier approach, and the differences in survival between two groups were compared using log-rank tests. A value of *P* < 0.05 was considered to indicate statistical significance.

## Results

### MicroRNA expression profiling revealed an increase in certain microRNAs, including miR-21-5p, in the serum EVs during experimental lung fibrosis in mice

**Fig. 1:**
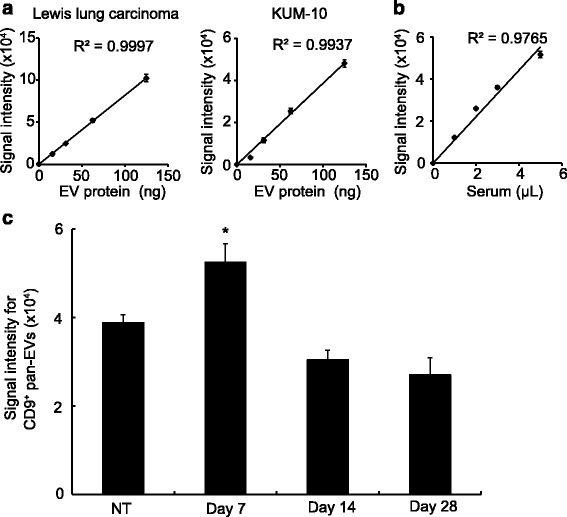
Circulating EVs increased during the acute phase of the experimental bleomycin-induced lung fibrosis model. **a** Detection of mouse CD9-positive pan-EVs by ExoScreen. Correlation between ExoScreen measurements for CD9-positive pan-EVs and EV protein concentration in a dilution series. EVs were purified from a mouse lung cancer cell line (Lewis lung carcinoma (LLC, left)) or a mouse mesenchymal cell line (KUM-10, right). Error bars are SEM; *n* = 3 for each condition. **b** Detection of circulating EVs in mouse sera. Correlation between ExoScreen measurements for CD9-positive pan-EVs and serum volume in a dilution series. Error bars are SEM; *n* = 3 for each condition. **c** Serum levels of CD9-positive pan-EVs in an experimental BLM-induced lung fibrosis model. Two microliters of serum from each mouse were used for the detection of EVs by ExoScreen. Note that the levels of pan-EVs were significantly higher in sera from mice at 7 days compared with those in non-treated (NT) mice. Error bars are SEM (*n* = 9 for each time point). The data were assessed for significance using the Kruskal-wallis test for multiple comparisons. *, *p* < 0.05 vs. NT mice

To identify the serum EV microRNAs that could serve as biomarkers for fibrotic lung diseases, we first utilized a mouse bleomycin-induced lung fibrosis model to investigate the differences in the expression of serum EV microRNAs compared to controls. We used the microRNA-expression profiles derived from the serum EVs rather than those from the total serum because previous reports [24, 25] and our pilot study data (Additional file 1: Figure S1) showed that analysing the microRNAs from the EV-rich serum fraction improved the reproducibility compared to the analysis of whole serum. We assumed that the amount of EVs was likely to influence the total amount of the EV microRNAs in the serum and therefore normalized the expression of the microRNAs by dividing these values by the amounts of the EVs. To analyse the EV quantities in the mouse serum and human samples, we utilized ExoScreen, which was recently introduced as a tool to investigate the EV levels and to profile the EV surface proteins in human samples [18]. We confirmed that ExoScreen successfully quantified the levels of the CD9-positive pan-EVs in both the cell culture supernatants and mouse serum samples, as previously reported for human samples [18] (Fig. 1a, b). We examined the changes in the levels of CD9-positive pan-EVs in the serum of mice that had been subjected to bleomycin-induced lung fibrosis. The levels of serum EVs were significantly increased on day 7 after administration relative to those in the untreated mice and returned to the basal levels on days 14 and 28 (Fig. 1c).

**Fig. 2:**
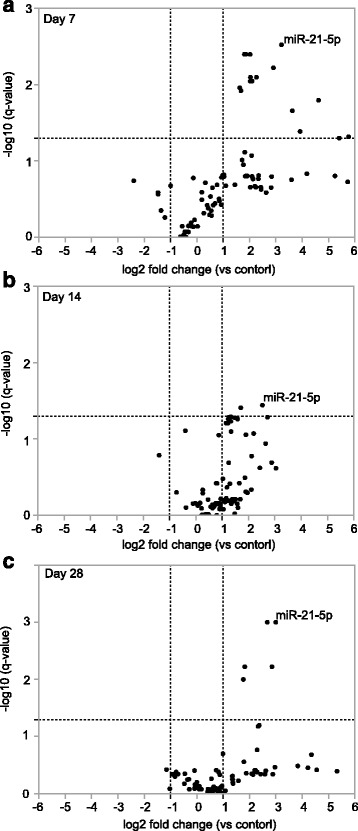
The microRNAs in the serum EVs increased significantly during experimental bleomycin-induced lung injury in mice. Volcano plots show the fold changes in the serum EV microRNAs compared with the non-treated controls in the bleomycin-induced lung fibrosis model at day 7 (**a**), day 14 (**b**) and day 28 (**c**) after administration of bleomycin. The horizontal dotted line indicates *q* = 0.05. The vertical dotted lines indicate fold changes = 2 or 0.5. The dots for miR-21-5p are indicated

To profile the changes in the microRNA expression in the serum EVs from the bleomycin-induced lung fibrosis model (histological analyses are shown in Additional file 1: Figure S2 [26]), we isolated the total RNA from the mouse serum EVs and performed quantitative real-time PCR array analyses (*n* = 3 for each group). The full Ct dataset corresponding to mouse microRNA PCR array data is provided as the online Additional file 2 (The full Ct dataset of mouse microRNA PCR array data.xlsx). We have also provided tables that show the average Ct values, including cel-miR-39 (spiked control), for each sample group (Additional file 1: Table S1), the average of the delta Ct values (target microRNA Ct–cel-miR-39 (spiked control) Ct) (Additional file 1: Table S2) and the pre-normalized fold-changes of serum EV microRNAs (Additional file 1: Table S3) in addition to the post-normalized fold-changes generated by dividing the pre-normalization values by the EV amounts in the serum samples (Additional file 1: Table S4) in the bleomycin-induced lung fibrosis model. We also examined the Ct values for miR-451a and miR-23a-3p to calculate a delta Ct value (miR-23a-3p - miR-451a), which is a previously reported indicator for erythrocyte miRNA contamination [27]. The delta Ct values (miR-23a-3p–miR-451a) for all of the mouse serum EV RNA samples used for the microRNA PCR array were less than five (Additional file 1: Table S5), which indicated that erythrocyte miRNA contamination was not significant in these samples. The PCR array analyses revealed that distinct microRNAs in the serum EVs significantly increased at each time point during the progression of lung fibrosis in the mice (Fig. 2). The fold-changes of the serum EV microRNAs that have been reported to be associated with the pathogenesis of lung fibrosis are shown in Table 1. Among the significantly increased microRNAs, miR-21-5p and miR-155-5p increased compared with the non-treated mice at every time point throughout the progression of the lung fibrosis (Table 2). Thus, these microRNAs could be considered as candidate biomarkers for lung fibrotic diseases.

**Table 1:** The fold-changes of the serum EV microRNAs that had been reported to be associated with the pathogenesis of lung fibrosis in the bleomycin-induced lung fibrosis model compared with the non-treated controls

Name	Day 7		Day 14		Day 28		Reference
	Fold change	q value	Fold change	q value	Fold change	q value	
Upregulated microRNAs in lung fibrosis							
miR-21-5p	9.29*	0.003	5.77*	0.036	7.97*	0.001	[19, 37–39]
miR-96-5p	ND		ND		ND		[42]
miR-145a-5p	0.68	0.715	2.35	0.647	0.81	0.554	[43]
miR-155-5p	4.28*	0.009	3.26*	0.039	6.37*	0.001	[44]
miR-195a-5p	1.79	0.314	1.40	0.976	1.13	0.828	[45]
Downregulated microRNAs in lung fibrosis							
miR-17-3p	12.33*	0.022	2.54	0.705	2.12	0.878	[46]
miR-17-5p	0.91	0.724	1.81	0.713	0.55	0.465	[46]
miR-18a-5p	1.90	0.375	2.59	0.622	1.46	0.920	[46]
miR-19a-3p	4.10*	0.008	2.51	0.059	1.99	0.199	[46]
miR-20a-5p	1.40	0.508	1.91	0.804	0.56	0.498	[46]
miR-29a-3p	1.81	0.333	1.55	0.805	0.52	0.451	[47]
miR-30d-5p	1.61	0.359	1.74	0.701	1.33	0.922	[48, 49]
miR-92a-3p	0.71	0.918	1.68	0.701	1.41	0.855	[46, 48]
miR-200a-3p	4.23	0.085	1.91	0.717	4.58	0.388	[50]
miR-200b-3p	4.29	0.159	1.20	0.515	2.55	0.546	[50]
miR-200c-3p^a^	3.41	0.112	3.03	0.797	2.55	0.554	[19, 50]

*ND* not detected (Ct > 35)

*q value < 0.05 compared with non-treated control

^a^microRNAs that had Ct > 30 in either group, which indicated that the expression levels were relatively low and could cause greater variations in the fold-change results

**Table 2:** Serum EV microRNAs that showed a significant* increase in the bleomycin-induced lung fibrosis model compared with non-treated controls

Day 7	Fold increase	Day 14	Fold increase	Day 28	Fold increase
miR-17-3p	12.33	**miR-21-5p**	**5.77**	**miR-21-5p**	**7.97**
**miR-21-5p**	**9.29**	**miR-155-5p**	**3.26**	miR-122-5p	7.24
miR-130b-3p	7.50	miR-106a-5p	2.27	**miR-155-5p**	**6.37**
miR-134-5p	4.81			miR-574-3p	3.53
**miR-155-5p**	**4.28**			miR-125b-5p	3.40
miR-19a-3p	4.10				
miR-122-5p	4.07				
miR-19b-3p	4.07				
miR-221-3p	3.64				
miR-106b-5p	3.51				
miR-22-3p	3.20				
miR-423-5p	3.09				

The expression levels of microRNAs were normalized to the amount of EVs in the mouse serum samples. The Ct values of these microRNAs were less than 30 indicating that the relative expression levels of these microRNAs were sufficiently high to evaluate for fold-changes without excessive variation. Both miR-21-5p and miR-155-5p (indicated by boldface) were significantly increased at every measured time point during the 28 days after bleomycin administration

*q value < 0.05 compared with non-treated control. The q value (false discovery rate) was calculated by the Benjamini-Hochberg procedure

**Fig. 3:**
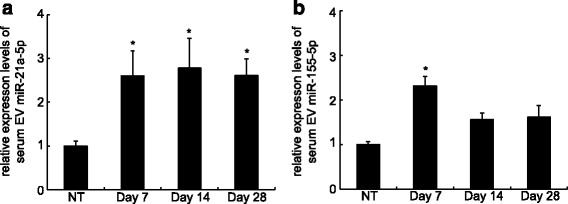
Increase of miR-21-5p and miR-155-5p in serum EVs during experimental bleomycin-induced lung injury. **a**, **b** Relative expression levels of both miR-21-5p (**a**) and miR-155-5p (**b**) are shown compared with non-treated control. The expression levels of microRNAs were normalized by the amount of EVs in the mouse serum samples. Error bars are SEM (*n* = 9 for each time point). The data were assessed for significance by Kruskal-wallis tests for multiple comparisons. *, *p* < 0.05 vs. non-treated control mice (NT)

Then, we performed conventional quantitative real-time PCR to examine and confirm the changes in the expression of EV miR-21-5p and miR-155-5p using a larger number of samples (*n* = 9) for each time point. The relative expression levels of these microRNAs were also normalized by dividing them by the EV amounts in the serum samples. The PCR analysis confirmed that EV miR-21-5p was significantly upregulated in the acute phase (at day 7) and in the later chronic/fibrotic phase (at days 14 and 28) (Fig. 3a). The levels of EV miR-155-5p were significantly increased in the acute phase (at day 7) but returned to the basal level in the chronic/fibrotic phase (Fig. 3b). These results showed that distinct microRNAs in the serum EVs increased over the course of the experimental lung fibrosis and that miR-21-5p, in particular, was upregulated significantly in both the acute phase and the chronic/fibrotic phase. This result suggested that serum EV miR-21-5p represents a likely candidate biomarker for fibrotic lung diseases, including IPF.

### Serum EV MiR-21-5p levels were elevated in IPF patients

Based on the results from the mouse lung fibrosis model, we hypothesized that the serum levels of EV miR-21-5p may be clinically associated with IPF. To test this hypothesis, we analysed the correlations between the serum EV miR-21-5p level and the changes in pulmonary function and survival in IPF patients.

A total of 41 IPF patients were enrolled in the prospective cohort study. The baseline characteristics of the study participants (21 healthy controls and 41 IPF patients) are shown in Table 3.

**Table 3:** The baseline characteristics of the healthy control subjects and the enrolled IPF patients

	Control (*n* = 21)	IPF (*n* = 41)
Sex, male, n (%)	16 (76)	32 (78)
Age, median (IQR)	69 (66–73)	72 (68–80)
Surgical lung biopsy undergo, n (%)	N.A.	15 (36)
Baseline % predicted VC, median (IQR)	109 (100–120)	81 (67–93)
Emphysematous lesion detected by CT, n (%) ^a^	N.A.	4 (10)
Biomarker, median (IQR)		
KL-6	N.A.	739 (557–1320)
SP-D	N.A.	207 (142–303)
LDH	N.A.	208 (183–247)
Smoking history		
Yes, n (%)	17 (80)	34 (82)
Therapy, n (%)	N.A.	
No treatment	N.A.	24 (58)
Pirfenidone	N.A.	10 (24)
Prednisolone	N.A.	8 (19)
Cyclosporine A	N.A.	2 (4)

The data are expressed as the median values (IQR) or n (%)

*N.A.* not acquired, *VC* vital capacity, *KL-6* Krebs von den Lungen-6, *SP-D* surfactant protein D

^a^ Goddard LAA score is ≥1

**Fig. 4:**
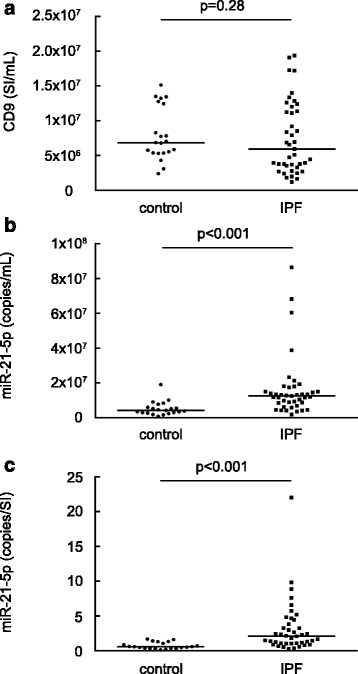
Serum EV miR-21-5p levels adjusted for the serum EV levels are increased in IPF patients. **a** The relative levels of human serum CD9-positive pan-EVs in healthy control subjects and IPF patients were determined using ExoScreen as signal intensities. **b** The expression levels of EV miR-21-5p in EVs isolated from 1 mL serum were examined by quantitative RT-PCR. **c** The expression of EV miR-21-5p normalized by dividing it by the relative EV amount is shown as the copy number of miR-21-5p divided by the signal intensity for CD9-positive pan-EVs in the serum samples. Differences between the groups were analysed using the Mann-Whitney *U* test

We measured the relative amounts of CD9-positive pan-EVs in the human serum samples using ExoScreen, in which the signal intensity (SI) value is relative to the total EV level [16]. Our pilot examination showed that the total RNA yields isolated from the serum EVs showed good correlation and regression with the relative levels of the serum CD9-positive EVs (signal intensities (SIs) for CD9 positive EVs) (Additional file 1: Figure S3). There was no significant difference in the total serum EVs between control and IPF subjects despite considerable variation even within the same subject groups (Fig. 4a). Subsequently, we evaluated the expression levels (copy numbers) of miR-21-5p contained in the EVs isolated from 1 mL of serum (miR-21-5p copy number/mL) (Fig. 4b). The levels of serum CD9-positive EVs were not significantly influenced by age or smoking history in our study subjects (Additional file 1: Figure S4). The expression levels of miR-21-5p in the EVs from 1 mL of serum were significantly greater in the IPF subjects than in the controls (Fig. 4b). Because we were interested in the net changes of miR-21-5p in the serum EVs, we then normalized the expression levels of the serum EV miR-21-5p by dividing them by the EV amount in each serum sample (miR-21-5p copy number/signal intensity for CD9-positive pan-EVs). The expression levels of the normalized serum EV miR-21-5p were also significantly greater in the IPF subjects than in controls (Fig. 4c). We also examined the expression levels of the normalized serum EV miR-21-5p in a set of patients with COPD, which is another type of chronic inflammatory pulmonary disease (Additional file 1: Table S6). There were no significant differences in the total serum EVs among control, COPD and IPF subjects (Additional file 1: Figure S5a). The expression levels of miR-21-5p in the EVs from 1 mL of serum in the COPD subjects were not significantly different from the controls but were significantly lower than those of the IPF subjects (Additional file 1: Figure S5b). The expression levels of the normalized serum EV miR-21-5p were also not significantly different from the controls but were significantly lower than those of the IPF subjects (Additional file 1: Figure S5c). There were no significant differences in the levels of the normalized serum EV miR-21-5p among the various GOLD stages in the COPD patients (Additional file 1: Figure S5d) and no significant correlations between the levels of serum EV miR-21-5p and severity of airway obstruction (Additional file 1: Figure S5e, f). Because the normalization step of dividing the expression levels by the EV amount improved variability of the measurements in the healthy control group (coefficient of variance; non-normalized levels of miR-21-5p: 0.755, normalized levels of miR-21-5p: 0.60), we used the normalized expression levels of serum EV miR-21-5p for the first of our subsequent analyses.

During the 30-month follow-up period, 11 patients died and nine patients were censored on the basis of failure to visit the hospital. We evaluated the correlations between the levels of serum EV miR-21-5p and the clinical parameters (Table 4) of the IPF subjects at the time of enrolment. The serum EV miR-21-5p levels were correlated with the rate of decline in the percent-predicted VC per 6 months in the IPF subjects (Table 4).

**Table 4:** The relationships between the normalized levels of EV miR-21-5p and the clinical variables in the 41 IPF patients

	miR-21-5p (copies/SI)	
	*r*_*s*_	*P*
% predicted VC	−0.02	0.86
KL-6	0.27	0.09
SP-D	0.11	0.51
LDH	0.17	0.29
Rate of decline in % predicted VC over 6 months	0.56	**<0.001**

*r*_s_: Spearman rank correlation coefficient. The correlations were calculated using the Spearman rank correlation test

Bold denotes *p* < 0.05

*VC* vital capacity, *DLco* carbon monoxide diffusing capacity, *KL-6* Krebs von den Lungen-6, *SP-D* surfactant protein D

### Serum EV miR-21-5p is a predictive biomarker for mortality in IPF patients

We calculated the hazard ratio (HR) using the Cox proportional hazards model to evaluate the association between the risk of death and clinical parameters, including serum EV miR-21-5p. In the univariate mortality analysis, age (HR, 1.09, 95 % CI, 1.001–1.20; *p* = 0.04) and EV miR-21-5p (HR, 1.15, 95 % CI, 1.04–1.25; *p* = 0.007) were associated with the risk of death during the following 30 months (Table 5). In the multivariate mortality analysis, EV miR-21-5p was still independently significant (EV miR-21-5p: HR, 1.12, 95 % CI, 1.003–1.24; *p* = 0.04) even after the adjustment for other variables. Moreover, we demonstrated that the association between miR-21-5p and mortality was significant even after adjusting for gender, KL-6, %VC and age (Table 6). These analyses suggest that the baseline level of serum EV miR-21-5p is a predictive biomarker for IPF prognoses.

**Table 5:** Univariate and multivariate Cox hazards model analyses for mortality during the 30-month follow-up period in the IPF patients

	Univariate		Multivariate	
	HR (95 % CI)	*P* value	HR (95 % CI)	*P* value
Sex				
Male	0.60 (0.17–2.78)	0.48		
Female	1.64 (0.35–5.75)	0.48		
Age	1.09 (1.001–1.20)^b^	**0.04**	1.04 (0.95–1.16)	0.31
% predicted VC	0.98 (0.95–1.01)^b^	0.31		
Emphysematous lesion detected by CT	2.20 (0.33–8.06)	0.35		
Rate (%) of decline in VC per 6 months	1.04 (0.98–1.09)^b^	0.11		
KL-6	0.99 (0.99–1.00)^b^	0.85		
SP-D	1.00 (0.99–1.00)^b^	0.41		
LDH	0.99 (0.97–1.00)^b^	0.23		
miR-21-5p (copies/SI)^a^ (per one copy/SI)	1.15 (1.04–1.25)^b^	**0.007**	1.12 (1.003–1.24)	**0.04**
CD9-positive EVs (×10^6^ SI/mL) (per 1 ×10^6^ SI/mL)	0.89 (0.75–1.02)^b^	0.10		

Bold represents *p* < 0.05

*HR* hazard ratio, *CI* confidence interval, *VC* vital capacity, *KL-6* Krebs von den Lungen-6, *SP-D*, surfactant protein D

^a^levels of serum EV miR-21-5p adjusted for the EV content in the serum samples

^b^A unit hazard ratio (hazard ratio per one unit change in each regressor) is shown

**Table 6:** The association between miR-21-5p of and mortality in IPF patients during 30 months using Univariate Cox Hazard model analyses with adjustment for each of the four parameters

The factor for adjustment	Univariate	
	HR (95 % CI)^a^	*P* value
Sex	1.16 (1.04–1.27)	**0.008**
Age	1.12 (1.003–1.24)	**0.04**
Emphysematous lesion detected by CT	1.19 (1.04–1.46)	**0.01**
KL-6	1.45 (1.15–1.83)	**0.002**
% predicted VC	1.16 (1.04–1.29)	**0.01**

Bold represents *p* < 0.05

*HR* hazard ratio, *CI* confidence interval, *KL-6* Krebs von den Lungen-6 *VC* vital capacity

^a^A unit hazard ratio (hazard ratio per one copy/SI in normalized serum EV miR-21-5p) is shown

**Fig. 5:**
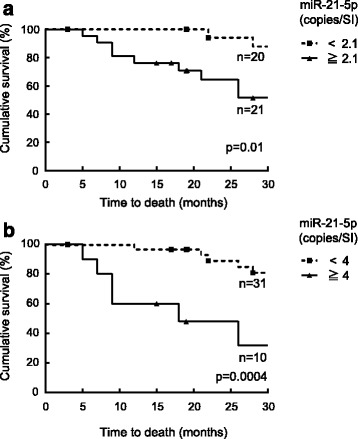
Kaplan-Meier analysis grouped by the normalized levels of the baseline serum EV miR-21-5p. **a** The solid line shows the group of IPF patients that had baseline serum EV miR-21-5p levels above the median level of EV miR-21-5p (2.1 copies/SI). The dashed line shows the group of IPF patients that had baseline serum EV miR-21-5p levels below the median level. The patients with the higher serum EV miR-21-5p levels showed higher mortality during the 30-month follow-up period (*p* = 0.01). **b** The solid line shows the group of IPF patients that had the top-quartile baseline serum EV miR-21-5p levels (≥4 copies/SI, *n* = 10). The dashed line shows the group of IPF patients that had baseline serum EV miR-21-5p levels below four copies/SI (*n* = 31). The patients who had serum EV miR-21-5p levels in the top-quartile showed higher mortality during the 30-month follow-up period (*p* = 0.0004). The dots represent cases that were censored because of failure to visit the hospital. The entire population (*n* = 41) contained nine censored patients

We then divided the IPF patients into two groups, those above and those below the median level of EV miR-21-5p (2.1 copies/SI) and performed a Kaplan-Meier analysis to investigate the difference between the survival curves of the patients with higher and lower serum EV miR-21-5p levels at the time of enrolment. The characteristics of each group are shown in Table 7. The proportion of males and the baseline KL-6 significantly differed between the two groups. As shown in Table 5, neither sex nor KL-6 was associated with the mortality during the 30-month period. We also examined whether there were differences between the blood cell counts of the patients with higher and lower levels of serum EV miR-21-5p. There were no significant differences in the numbers of the white blood cells, red blood cells, platelets, neutrophils, lymphocytes, monocytes, eosinophils or basophils (Additional file 1: Table S7). We also investigated whether there were correlations between the levels of EV miR-21-5p and the numbers of each type of blood cells and showed that there were no significant correlations between the levels of EV miR-21-5p and the blood cell counts (Additional file 1: Figure S6). There was no significant difference in the number of censored patients between the higher and lower groups (five vs. four patients, respectively). The Kaplan-Meier analysis showed that the survival of the patients with higher serum EV miR-21-5p levels was significantly worse than that of the patients with lower EV miR-21-5p levels. Nine (42 %) of the patients with higher baseline serum EV miR-21-5p levels died, whereas two (10 %) of the patients with lower EV miR-21-5p levels died during the 30-month follow-up period (Fig. 5a). We also performed the Kaplan-Meier analysis between the group of ten patients with levels of EV miR-21 that were in the top quartile (more than four copies/SI) and the group of 31 patients for whom the levels of EV miR-21 were less than four copies/SI (Fig. 5b). This analysis also clearly showed that the survival of the top quartile group was worse than that of the group with the lower levels of EV miR-21. These analyses clearly showed that the survival of the patients with high levels of serum EV miR-21 was significantly worse than that of the patients with low levels of serum EV miR-21.

**Table 7:** Characteristics of the two groups into which IPF patients were divided according to the median of the normalized levels of serum EV miR-21-5p

	Normalized miR-21-5p (copies/SI)		*P* value
	<2.1	≥2.1	
	(*n* = 20)	(*n* = 21)	
Age, yr	71 (65–77)	75 (71–81)	0.15
Sex, male, n (%)	19 (95)	13 (61)	**0.02**
Smoking history			
Yes, n (%)	18 (90)	16 (76)	0.4
Baseline VC (%)	78 (69–104)	81 (62–91)	0.48
Emphysematous lesion detected by CT, n (%)	2 (10)	2 (10)	0.96
KL-6	645 (540–982)	1040 (706–1921)	**0.04**
SP-D	211 (141–277)	202 (140–320)	0.77
LDH	207 (183–215)	227 (177–280)	0.18

The data are expressed as n (%) or median (IQR). The differences in age, % VC, or other serum markers between the two groups were analysed by the Mann-Whitney *U* test. The comparison of gender, smoking history and emphysema between the two groups was analysed using the Fisher exact test

Bold represents *p* < 0.05

*VC* vital capacity, *KL-6* Krebs von den Lungen-6, *SP-D* surfactant protein D

### Analyses of the benefit of normalization by the EV amount of each serum sample in the correlation between the non-normalized levels of Serum EV MiR-21-5p and clinical parameters, risk of death and survival

We also performed an analysis of the correlation between the non-normalized EV miR-21-5p copy numbers and the clinical parameters as well as their association with the risk of death and Kaplan-Meier analysis. The levels of the non-normalized EV miR-21-5p copy numbers in the serum were also correlated with the rate of decline in percent-predicted VC per 6 months and the levels of serum SP-D (Additional file 1: Table S8). The levels of the non-normalized miR-21-5p copy numbers were also associated with the risk of death during the following 30 months (Additional file 1: Table S9). However, the Kaplan-Meier analysis showed that there was no significant difference between the survival curves for the patients with serum EV non-normalized miR-21-5p copy numbers that were higher versus lower than the median (Additional file 1: Table S10 and Figure S7a). We also performed the Kaplan-Meier analysis to compare the group of 10 patients with levels of EV miR-21 in the top quartile (more than 1.75 × 10^7^ copies/mL) and the group of 31 patients in whom the levels of EV miR-21 were less than 1.75 × 10^7^ copies/mL. The results of this analysis showed that there was a significant difference between the mortality rates for those in the top quartile and the remaining subjects (Additional file 1: Figure S7b). Then, we performed a receiver-operating-characteristic (ROC) curve analysis to evaluate whether the normalized miR-21-5p copy numbers were more predictive of death of IPF patients than the non-normalized levels of the serum EV miR-21-5p (Additional file 1: Figure S7c). The ROC analysis indicated that the area under the curve (AUC) for the normalized levels of serum EV miR-21-5p was 0.857 (95 % CI = 0.671–0.955). The AUC for non-normalized levels of EV miR-21-5p was lower (0.788; 95 % CI = 0.541–0.921) than that for normalized levels, though the difference was not statistically significant (*P* = 0.51). Altogether, although the non-normalized EV miR-21-5p levels were also associated with disease progression and the risk of death, our data may suggest that the normalized serum EV miR-21-5p levels might be the better predictor of mortality in IPF patients.

### The changes of levels of serum EV miR-21-5p after treatment with Pirfenidone

In four out of the ten patients who were administered Pirfenidone, we examined the levels of the serum EV miR-21-5p in serum samples at 6 months and/or 12 months after registration and starting the treatment (Additional file 1: Figure S8a). Although the sample size is small, this examination showed a tendency for the patients who seems to be responsive to Pirfenidone (patients IPF 11 and 15) to have levels of serum EV miR-21-5p that decreased or were maintained at low levels at 6 and 12 months after starting the treatment whereas the non-responder subjects (patients IPF 10 and IPF 40) showed elevated levels of serum EV miR-21-5p at 6 and/or 12 months (Additional file 1: Figure S8b). These data suggest that levels of serum EV miR-21-5p might be useful as biomarkers of treatment responsiveness.

## Discussion

This study first demonstrated that the expression profile of the microRNAs within the EVs in circulating blood was significantly changed in a mouse bleomycin-induced lung fibrosis model. Among the microRNAs that showed an altered expression during lung fibrosis, serum EV miR-21-5p expression was significantly upregulated in both the acute phase and the later chronic/fibrotic phase. In human subjects, the levels of serum EV miR-21-5p after adjustment for the quantity of EVs were significantly increased in the IPF patients. Moreover, the baseline levels of the serum EV miR-21-5p were significantly associated with the disease progression (the decline in the percent-predicted VC) and mortality. This is the first report to show that serum EV microRNA could be a prognostic biomarker for IPF.

The circulating microRNAs in the whole serum have been investigated by many researchers in their efforts to identify useful candidate biomarkers for various diseases [28, 29]. However, previous reports and the data from our pilot study (Additional file 1: Figure S1) showed that analysing the microRNAs from the EV-rich serum fraction improved the reproducibility compared to the analysis of whole serum [24, 25]. Furthermore, there is no clear consensus in the research community as to the appropriate normalization control for microRNA expression profiling in serum samples. Moreover, because the components of the EVs and their secretion dynamics vary according to their cellular origin and environment [30], it is possible that the data obtained from an EV analysis would reflect the condition of chronic diseases, including fibrosis. Therefore, we focused on the EV-associated microRNAs in the serum and examined their changes during lung fibrosis. We hypothesized that the mouse model could be suitable for a comprehensive assay using PCR arrays due to its consistency and simple background relative to human subjects, although the mouse model does not ideally mimic the human disease. Therefore, we first analysed the changes in serum EV microRNAs in a mouse model of bleomycin-induced lung fibrosis to identify candidate microRNAs for biomarkers of fibrotic lung diseases. The PCR array assays identified the EV microRNAs that were changed in the mouse model. Among these microRNAs, additional confirmatory quantitative PCR assays revealed that miR-21-5p was upregulated in both the acute and chronic/fibrotic phase, which suggested that only serum EV miR-21-5p was changed throughout lung fibrosis and could serve as a biomarker for human fibrotic lung diseases, including IPF. In the human subjects, we examined the levels of miR-21-5p in the serum EVs and the levels of EVs in serum samples. Our data showed that, although there were no significant differences among healthy controls, COPD patients and IPF patients, the EV levels in serum were highly variable, even within the same subject group. We assumed that the levels of EVs would influence the levels of the microRNAs in the samples. Moreover, we were interested in the net change in the microRNA content of the EVs, rather than the total amount of the microRNA in the serum EV samples, because the change in the content of individual species, rather than the total content, reflects the condition of cells or tissues during disease. Therefore, we then attempted to normalize the expression levels of the serum EV miR-21-5p by dividing them by the EV amount in each serum sample (miR-21-5p copy number/signal intensity for CD9-positive pan-EVs). This normalization step also improved the variability in the measurements in the healthy control group. On the basis of these results, we used the normalized levels of serum EV miR-21-5p for our initial analyses.

The correlation analyses between the serum EV miR-21-5p and clinical parameters indicated that both normalized and non-normalized serum EV miR-21-5p levels were correlated with the rate of decline in the percent-predicted VC over 6 months. Furthermore, we demonstrated that the miR-21-5p baseline levels could predict the mortality of IPF patients during the 30-month follow-up period. Our data suggested that the levels of serum EV miR-21-5p at baseline are predictive of long-term mortality in IPF patients and can also predict the short-term disease progression in terms of the decline in lung function.

Two recent reports have analysed the expression of microRNAs in the serum samples of IPF patients [31, 32]. These two reports showed that the miR-21-5p in whole serum was increased in the IPF patients compared to the healthy controls, which is consistent with our findings from the serum EV microRNA analyses. However, in our prospective cohort study, we first clearly demonstrated that the baseline levels of serum EV miR-21-5p were significantly correlated with the disease progression (a decrease in predicted % VC) and were associated with mortality during the 30-month follow-up period. Our study is the first report to suggest that the microRNA in the serum EVs could be a promising candidate for a prognostic biomarker in patients with IPF.

Our study has not elucidated the mechanism for the increase in the serum EV miR-21-5p in IPF patients. Accumulating evidence has suggested that miR-21-5p plays a vital role in various biological responses and pathological processes [33–35]. A study that used miR-21 gene-targeted mice clearly showed that miR-21-5p targeted tumour suppressor genes, including *spry1*, *pten*, and *pdcd4* [33]. The expression of miR-21-5p is also regulated at the post-transcriptional level by the TGF-β family of proteins and their downstream signal transducers, the SMADs [36], which are key regulators in the pathogenesis of fibrosis. Our previous reports and those of others have shown that miR-21-5p is increased in whole-lung samples from both bleomycin-induced mouse models of lung fibrosis and human patients with IPF [19, 37–39]. MiR-21-5p targets an inhibitory SMAD called SMAD7, and administration of miR-21-5p antisense probes attenuates the severity of the bleomycin-induced fibrosis by blocking the positive feedback loop of TGF-β signalling [38]. MiR-21-5p is also expressed in lung epithelial cells, and increased miR-21-5p expression was observed in the lungs of patients with IPF [19]. It is possible that the increased levels of serum EV miR-21-5p reflect the conditions of fibrotic lung diseases, including IPF, in which TGF-β signalling is one of the most relevant signalling pathways. However, miR-21-5p expression is also regulated by other factors that are involved not only in fibrosis but also in cell proliferation and inflammation. It is, therefore, likely that the reason for the increases in the levels of serum EV miR-21-5p in IPF patients is more complicated.

There are limitations to this study. First, the sample size for the human study was relatively small. This may explain why this study did not show that factors, including male gender, were risk factors for death, although these factors have been reported as independent risk factors for disease progression [40, 41]. To confirm our observation, another cohort study with a larger sample size would be needed. Next, we isolated (or concentrated) the EVs from the sera using a reagent (Total Exosome Isolation reagents) and then examined both the amount of EVs using Exoscreen and the microRNA expression profiles, followed by the analyses of the microRNA expression profiles after normalization to the amount of EVs. However, the isolation reagent contains polymers that sequester the water molecules to force the less-soluble components, such as vesicles, to be precipitated. It is, therefore, possible that other vesicles, particles and protein aggregates could have contaminated the precipitate that contains the EVs. This isolation reagent may therefore not be the ideal method for the isolation of pure EVs. Exosomal biomarkers have the advantage of being more specific and stable compared to other biomarkers from the biological fluid. However, it will be very important to increase the efforts to establish a standardized method for the isolation of pure EVs and the microRNA inside these EVs for both disease prediction and pathogenesis elucidation. Third, Exoscreen seems to be a better available method to evaluate the amount of EVs on the basis that we observed that the total yield of RNA (mainly, small RNAs; Additional file 1: Figure S3) isolated from the serum EVs showed a good correlation with the relative levels of serum CD9-positive EVs (signal intensities (SIs) for CD9 positive EVs). However, it is possible that the EVs for which the expression is low or is downregulated in the pathological conditions may cause discrepancy between signal intensities (SIs) for CD9 positive EVs and the actual amount of EVs. Because there is no established method to count the absolute number of EVs or measure the absolute amount of EVs, it is difficult to solve this possible discrepancy at the present. We also think that it is also important to establish the method to measure the absolute number or amount of EVs in biological fluid samples for normalization. Moreover, to explore the method to count and isolate the specific EVs originated form specific types of cells (for example, epithelium or endothelium) or the cells under pathological conditions is thought to be useful for both establishing biomarkers and understanding pathogenesis of various diseases. Final, we first used the normalized levels of serum EV miR-21-5p for our analyses because we were interested in the net change in the microRNA contents of the EVs. However, although this normalization step improved the variability in the measurements in the healthy control group and some of the analysed results suggested that the normalization might have improved the ability to predict the mortality of the IPF patients (Additional file 1: Figure S7), it is also true that the benefit of this normalization seems to have been minimal in this study (Additional file 1: Table S8 and S9). To confirm the benefit of the normalization, another cohort study with a larger sample size and improvements in both the EV isolation and EV measurement would be needed.

## Conclusions

Our comprehensive analysis of the expression of microRNAs in serum EVs in a mouse lung fibrosis model revealed miR-21-5p as a potential prognostic biomarker of IPF. In our clinical studies, the levels of serum EV miR-21-5p adjusted by the EV amount significantly predicted both the disease progression and mortality. EV miR-21-5p could be useful to distinguish patients in need of intensive therapies, including novel therapies.

## Additional files

Additional file 1: Online supplemental material. (PDF 9 mb) Additional file 2: The full Ct dataset corresponding to mouse microRNA PCR array data. (XLSX 20 kb)

## Abbreviations

CI: Confidence interval
COPD: Chronic obstructive pulmonary disease
CT: Computed tomography
DLco: Diffusing capacity of the lung for carbon monoxide
EV: Extracellular vesicle
GOLD: Global Initiative on Obstructive Lung Disease
HR: Hazard ratio
IPF: Idiopathic pulmonary fibrosis
IQR: Interquartile range
KL-6: Krebs von den Lungen
rs: Spearman rank correlation coefficient
SEM: Standard error of the mean
SI: Signal intensity
SP-D: Surfactant protein D
VC: Vital capacity
