# Supplementary material for: Serum extracellular vesicular miR-21-5p is a predictor of the prognosis in idiopathic pulmonary fibrosis
Source: Respir Res. 2016 Sep 5;17(1):110. doi: 10.1186/s12931-016-0427-3 (PMC5011900; doi:10.1186/s12931-016-0427-3)

Serum extracellular vesicular miR-21-5p is a predictor of the prognosis in idiopathic pulmonary fibrosis

Tomonori Makiguchi, Mitsuhiro Yamada, Yusuke Yoshioka, Hisatoshi Sugiura, Akira Koarai, Shigeki Chiba, Naoya Fujino, Yutaka Tojo, Chiharu Ota, Hiroshi Kubo, Seiichi Kobayashi, Masaru Yanai, Sanae Shimura, Takahiro Ochiya and Masakazu Ichinose

Online supplemental material

Methods for the supplemental data

Reproducibility of the analyses to measure the levels of microRNAs in the serum EVs.

Male 8-week-old C57BL/6J mice were administered 1 mg/kg of bleomycin hydrochloride (n = 5) or saline (n = 5) by intrabronchial instillation. Blood samples were collected via the vena cava on day 14 after instillation. The blood samples were incubated at room temperature for 30 minutes and then at 4 °C overnight. The serum samples were harvested by centrifugation (at 1,600 g, 4 °C for 10 minutes and 10,000 g, 4 °C for 10 minutes). The total RNA from 100 µL of serum was prepared using the miRNeasy mini kit (QIAGEN, Hilden, Germany) according to the manufacturer's instructions. The total RNA from the serum EVs was prepared using Total Exosome Isolation reagent (Thermo Fisher Scientific, Waltham, MA, USA). Briefly, 100 µL of serum were mixed with 20 µL of the Total Exosome Isolation reagent and incubated for 30 minutes at 4 °C. The samples were then centrifuged at room temperature at 10,000 g for 10 minutes. The supernatants were discarded, and the EV pellets were resuspended in 100 µl of PBS. The EV suspensions were used for further examination. The RNA was extracted using the miRNeasy mini kit (QIAGEN, Hilden, Germany). The total RNA samples were independently extracted twice from the same serum sample with or without EV enrichment using the Total Exosome Isolation reagent. The miScript Reverse Transcription Kit (QIAGEN) was used for reverse transcription of microRNAs into cDNA. miR-21a-5p and miR-155-5p were quantified by quantitative PCR using the miScript Primer Assay (QIAGEN).

The measurements of the microRNA expression of the same sample were compared between the two independent experiments.

Measurement of RNA yields from serum EVs

An Agilent 2100 Bioanalyzer (Agilent Santa Clara, CA, USA) and an RNA 6000 Pico Kit (Agilent) were used to quantify the RNA concentration and examine the types of RNA that were present in the RNA samples extracted from the serum EVs. We calculated the amounts of the RNAs in the EV samples derived from 1 mL of serum based on the measured concentrations of the RNA solution extracted from the serum EVs. We then determined whether there was a correlation between the yields of the RNA that was isolated from the serum EVs and the relative levels of the serum CD9-positive EVs (signal intensities (SIs) for CD9-positive EVs).

Subjects and specimens of COPD patients

This study was approved by the ethics committees at Tohoku University School of Medicine, Japanese Red Cross Ishinomaki Hospital. All subjects provided written informed consent.

Human peripheral blood was obtained from patients at Japanese Red Cross Ishinomaki Hospital (Ishinomaki, Japan). Serum was obtained by centrifuging these specimens, aliquoted, and stored at -80 ºC until used in the analyses of the serum EVs. Twenty-four patients with COPD (n =6 for each GOLD stage) were included (supplemental Table S6).

Histological assessment of mouse lungs

The mouse lungs were inflated with 10% formalin through a tracheal catheter at a transpulmonary pressure of 15 cm H_2O. The fixed lungs were embedded in paraffin and sectioned by standard methods. The lung specimens were stained with Elastica-Masson (EM) and examined using a light microscope to detect the histological changes. We examined 10 fields of view from each section and analysed them in a blinded manner. The severity of fibrosis was assessed using the modified Ashcroft scale as previously described [26].

Figure legends for online supplemental figures

Fig. S1 Reproducibility of the analyses to measure the levels of microRNAs in serum EVs. **a** Scatter plots comparing the relative levels of miR-21 (upper) or miR-155 (lower) in two independent experiments from total mouse serum and the EV-rich fraction. Total serum extracted in the first versus the second experiment (left), and EVs extracted in the first versus the second experiment (right). **b** Pearson's pairwise correlations of the relative levels of miR-21a-5p (upper) or miR-155-5p (lower) from total human serum and the EV-rich fraction. Note the analyses that used total RNA extracted from the serum EV samples were more reproducible than those that used total RNA from total serum. n = 10 for each independent experiment.

Fig. S2 Histological examination of the lung tissues from mice subjected to the experimental bleomycin-induced lung injury model. **a** and **b** Representative images of the lung tissues from a non-treated control mouse (**a**) and a bleomycin-induced lung injury model mouse at 28 days after administration (**b**). **c** Quantitative assessment of the severity of the fibrosis during bleomycin-induced lung injury. The histological assessment was performed using the modified Ashcroft score. n = 9 in each group.

Fig. S3 RNA yields from serum EVs. **a** A representative electropherogram of RNA isolated from the human serum EV samples using the Total Exosome Isolation Reagent (Thermo Fisher Scientific) showing the RNA pattern analysed with the Agilent RNA 6000 Pico Assay. Small RNAs (< 200 nt) were the main species that were detected whereas the 18S and 28S ribosomal RNAs were not detected, which indicated very little contamination with cellular RNAs. **b** Regression analysis between the relative levels of serum CD9-positive EVs (signal intensities (SIs) for CD9-positive EVs) and the concentration of the RNA isolated from the serum EV

samples (n = 20).

Fig. S4 The levels of serum CD9-positive EVs in our study subjects were not significantly influenced by age or smoking history. **a** and **b** Scatter plots for age and the levels of serum CD9-positive EVs in healthy control subjects (**a**; n = 21) or in all studied subjects including control and IPF subjects (**b**; n = 62). There were no significant correlations between these parameters in either the control subjects or all studied subjects. The correlations were analysed using Spearman rank correlation. **c** and **d** The levels of serum CD9-positive EVs in the smokers or never smokers among the healthy control subjects (**c**; n = 21) or all studied subjects including control and IPF subjects (**d**; n = 62). There were no significant differences between the smokers and never smokers in either the control subjects or all studied subjects. Each box plot shows the minimum, lower quartile, median, upper quartile and maximum of the values of each group. Outliers are plotted as individual points outside the boxes.

Fig. S5 The serum EV miR-21-5p levels adjusted for the serum EV levels were not increased in the COPD patients. **a** The relative levels of human serum CD9-positive pan-EVs in healthy control subjects (n = 21), COPD (n = 24) and IPF patients (n = 41) were determined as the signal intensities from the ExoScreen analysis. **b** The expression levels of EV miR-21-5p in the EVs isolated from 1 mL of serum were examined using quantitative RT-PCR. **c** The expression of EV miR-21-5p normalized by dividing it by the relative EV amount. The values shown are the copy numbers of miR-21-5p divided by the signal intensities for the CD9-positive pan-EVs in the serum samples. The differences between the groups were analysed using the Steel-Dwass test. **d** Comparison of the levels of normalized EV miR-21-5p among GOLD stages of COPD patients (n = 6 for each stage). There were no significant differences among the GOLD stages. **e** and **f**

Scatter plots for the levels of normalized EV miR-21-5p versus FEV_1 (%pred) (**e**) or FEV_1/FVC (**f**) in the COPD patients (n = 24). There were no significant correlations between these parameters in COPD patients. The correlations were analysed using Spearman rank correlation.

Fig. S6 Scatter plots for the levels of normalized EV miR-21-5p and the numbers of white blood cells (WBCs, **a**), red blood cells (RBCs, **b**), platelets (**c**), neutrophils (**d**), lymphocytes (**e**), monocytes (**f**), eosinophils (**g**) and basophils (**h**) in IPF patients (n = 41). There were no significant correlations between the levels of normalized EV miR-21-5p and the blood cell counts for any type of cells in the IPF patients. The correlations were analysed using Spearman rank correlation.

Fig. S7 Kaplan-Meier analysis grouped by the non-normalized levels of the baseline serum EV miR-21-5p. **a** The solid line shows the group of IPF patients that had baseline non-normalized serum EV miR-21-5p levels above the median level of EV miR-21-5p ($1.25\ x10^7$ copies/mL). The dashed line shows the group of IPF patients that had baseline non-normalized serum EV miR-21-5p levels below the median level. There was no significant difference in mortality between these two groups during the 30-month follow-up period. **b** The solid line shows the group of IPF patients that had baseline non-normalized serum EV miR-21-5p levels in the top quartile ($\geq 1.75\ x10^7$ copies/mL, n =10). The dashed line shows the group of IPF patients that had baseline serum EV miR-21-5p levels below $1.75\ x10^7$ copies/mL (n = 31). The patients with the top-quartile serum EV miR-21-5p levels showed higher mortality during the 30-month follow-up period ($p = 0.0004$). The dots represent cases that were censored on the basis of failure to visit the hospital. The entire population (n=41) contained 9 censored patients.

Fig. S8 The levels of serum EV miR-21-5p after treatment with Pirfenidone. **a** The list of IPF patients who were treated with Pirfenidone and whose serum samples were analysed at 6 months and/or 12 months after starting the treatment. **b** The changes in the levels of EV miR-21-5p during Pirfenidone treatment.

Online supplemental table

Table S1 The average Ct values from the MicroRNA PCR array analysis of the mouse serum EVs from the control mice and the bleomycin-induced lung fibrosis model mice.

	Average Ct				Standard Deviation			
NAME	control	Day 7	Day 14	Day 28	control	Day 7	Day 14	Day 28
let-7a-5p	25.02	24.82	24.70	26.35	0.82	0.94	1.02	0.57
let-7c-5p	25.26	25.32	23.72	25.74	0.38	0.63	1.18	1.08
miR-1a-3p	30.18	24.99	30.05	27.39	1.04	0.54	0.62	0.76
miR-7a-5p	30.23	28.45	28.72	29.89	1.23	0.99	1.21	1.32
miR-9-5p	34.25	34.50	33.17	34.66	0.61	0.25	0.16	0.59
miR-10a-5p	29.13	27.50	27.50	27.28	1.60	1.33	0.78	1.62
miR-10b-5p	27.94	29.51	27.65	28.98	0.34	0.30	0.82	1.09
miR-15a-5p	24.65	25.69	24.70	24.79	1.07	0.61	0.84	1.63
miR-15b-5p	22.99	23.97	22.10	23.93	0.66	1.13	0.97	1.41
miR-16-5p	19.56	20.44	17.99	20.51	1.43	0.47	0.95	1.26
miR-17-3p	29.19	26.13	27.79	29.14	1.33	0.38	0.75	0.78
miR-17-5p	22.56	23.27	21.65	24.47	0.89	1.32	1.41	0.78
miR-18a-5p	25.39	25.04	23.96	25.89	1.16	1.11	0.88	1.50
miR-19a-3p	24.80	23.30	23.41	24.84	0.18	0.21	0.24	0.14
miR-19b-3p	24.67	23.19	23.28	24.98	0.17	0.23	0.22	0.01
miR-20a-5p	22.74	22.83	21.75	24.61	1.20	0.47	0.71	0.07
miR-21a-5p	22.19	19.60	19.50	20.39	0.27	0.16	0.25	0.06
miR-22-3p	23.78	22.63	22.49	24.22	0.17	0.24	0.19	0.20
miR-23a-3p	22.80	22.78	21.93	21.55	0.36	0.69	0.24	1.54
miR-24-3p	23.60	23.13	21.11	22.22	0.22	0.76	0.84	1.44
miR-25-3p	22.83	22.47	20.91	22.53	0.08	0.72	1.00	1.39
miR-26a-5p	26.20	25.79	25.09	27.32	0.35	0.77	0.90	1.02
miR-26b-5p	23.79	26.78	23.27	25.85	0.61	1.32	0.87	1.25
miR-27a-3p	22.00	22.53	21.77	23.04	1.42	1.25	0.61	0.78
miR-29a-3p	21.88	21.60	21.19	23.86	1.11	0.85	1.22	1.10
miR-30d-5p	25.56	25.44	24.70	26.19	0.71	1.19	0.98	1.27
miR-30e-5p	23.36	25.14	21.85	24.28	1.19	1.10	0.95	1.08
miR-31-5p	30.33	28.27	28.58	30.78	1.30	1.34	0.91	1.00
miR-34a-5p	29.09	26.82	26.91	28.05	1.18	0.93	0.24	0.94
miR-34c-5p	34.28	32.43	32.98	33.74	0.17	1.04	0.39	1.46
miR-92a-3p	22.63	23.70	21.82	23.17	0.15	0.82	1.29	1.03
miR-93-5p	23.66	23.68	20.72	23.84	0.99	1.05	0.66	0.76
miR-96-5p	ND	ND	ND	ND	ND	ND	ND	ND
miR-100-5p	29.10	27.64	27.75	28.01	0.17	1.00	0.80	1.09
miR-103-3p	26.65	26.13	26.38	28.16	1.06	0.63	0.72	1.18
miR-106a-5p	23.64	23.95	22.10	23.81	0.88	0.64	0.54	1.46
miR-106b-5p	24.38	23.26	23.15	24.70	0.22	0.13	0.05	0.06
miR-107-3p	31.20	27.85	30.19	31.42	0.73	0.44	0.43	0.63
miR-122-5p	27.14	25.80	25.82	25.32	0.23	0.10	0.13	0.16
miR-124-3p	32.28	30.94	29.50	31.45	0.47	0.64	0.32	1.00

miR-125b-5p	27.60	28.50	26.67	27.10	0.25	0.16	0.07	0.03
miR-126a-3p	24.78	25.57	22.52	24.46	0.31	1.12	0.44	1.32
miR-128-3p	24.98	24.93	23.82	26.37	1.29	1.56	1.33	0.92
miR-130b-3p	28.68	26.52	27.08	29.30	0.25	0.15	0.22	0.17
miR-133a-3p	32.82	28.76	31.20	29.64	0.44	0.49	0.99	1.09
miR-133b-3p	34.38	29.54	33.54	30.09	0.67	0.39	0.84	1.35
miR-134-5p	28.60	26.97	27.21	29.12	0.21	0.22	0.10	0.11
miR-141-3p	31.71	28.10	29.70	30.46	0.23	0.81	1.24	0.41
miR-143-3p	25.47	27.52	24.72	25.61	0.78	0.49	0.68	1.29
miR-145a-5p	25.42	26.56	24.13	26.77	1.47	0.78	1.35	0.58
miR-146a-5p	26.44	28.38	25.75	26.95	0.59	1.08	1.23	1.27
miR-148a-3p	26.10	26.29	24.57	25.78	0.91	0.27	1.49	1.26
miR-150-5p	23.85	25.05	23.49	24.96	0.83	1.33	0.91	0.81
miR-155-5p	29.45	27.81	27.69	27.94	0.14	0.24	0.20	0.05
miR-181a-5p	28.35	27.20	29.68	30.53	0.52	0.57	0.50	1.10
miR-181b-5p	28.68	27.39	26.97	30.18	0.04	1.01	0.90	0.95
miR-184-3p	ND	ND	ND	ND	ND	ND	ND	ND
miR-191-5p	23.26	22.00	22.07	23.55	0.77	0.75	0.74	1.49
miR-192-5p	26.35	26.49	25.72	26.84	0.57	1.30	0.91	0.99
miR-193a-3p	31.98	26.83	29.81	28.67	1.09	1.08	1.00	0.81
miR-195a-5p	20.29	20.02	19.74	21.15	1.35	0.84	0.97	1.10
miR-196a-5p	32.38	31.92	32.70	32.46	0.74	0.63	1.44	0.84
miR-199a-5p	32.22	30.46	32.04	31.04	0.45	1.12	0.42	1.13
miR-200a-3p	29.96	28.46	28.97	28.81	0.63	0.51	1.48	0.82
miR-200b-3p	29.04	27.51	28.72	28.72	0.95	0.66	0.36	0.61
miR-200c-3p	30.19	28.99	28.53	29.88	0.98	0.88	1.04	0.89
miR-203-3p	26.70	27.49	27.38	28.52	0.68	1.15	0.89	0.69
miR-204-5p	33.18	31.93	32.22	32.09	1.48	0.31	0.74	1.35
miR-205-5p	27.53	28.23	27.86	26.27	0.20	0.06	0.24	0.29
miR-206-3p	32.93	29.90	32.01	31.00	0.82	0.83	1.14	0.84
miR-210-3p	26.51	26.90	26.06	27.45	0.45	0.39	0.07	0.85
miR-211-5p	33.92	32.36	32.79	34.20	0.82	0.87	0.93	0.13
miR-214-3p	28.96	28.09	28.92	27.67	0.57	1.25	0.75	0.28
miR-215-5p	26.18	28.24	24.98	27.95	0.94	0.31	0.83	0.54
miR-221-3p	27.98	26.69	26.44	28.38	0.25	0.15	0.22	0.09
miR-222-3p	25.78	26.16	23.82	26.15	0.55	0.49	0.35	1.03
miR-223-3p	23.75	23.76	22.23	24.87	1.18	1.02	1.26	1.21
miR-224-5p	32.15	30.28	31.20	31.40	0.75	1.31	1.05	0.34
miR-296-5p	30.18	25.50	27.07	28.63	1.37	1.14	0.75	1.33
miR-375-3p	28.28	28.94	25.57	27.95	0.54	0.59	0.64	1.34
miR-376c-3p	32.47	30.22	31.62	28.96	1.33	1.19	0.43	1.40
miR-423-5p	28.50	27.47	27.29	28.73	0.25	0.23	0.18	0.02
miR-499-5p	34.15	32.48	32.20	32.57	1.23	1.03	0.82	0.70
miR-574-3p	28.82	29.82	27.55	28.24	0.22	0.14	0.20	0.02
cel-miR-39-3p	13.90	14.91	13.49	14.42	0.14	0.20	0.14	0.09

ND: not detected (Ct > 35).

Table S2 The average delta Ct values (target microRNA Ct - cel-miR-39 Ct) from the MicroRNA PCR array analysis of the mouse serum EVs from the control mice and the bleomycin-induced lung fibrosis model mice.

	Average delta Ct				Standard Deviation			
NAME	control	Day 7	Day 14	Day 28	control	Day 7	Day 14	Day 28
miR-16-5p	5.66	5.54	4.51	6.08	1.52	0.39	0.92	1.28
miR-195a-5p	6.39	5.12	6.26	6.72	1.27	0.91	1.05	1.13
miR-29a-3p	7.98	6.69	7.71	9.44	1.01	0.77	1.27	1.12
miR-27a-3p	8.10	7.63	8.29	8.62	1.33	1.27	0.67	0.87
miR-21a-5p	8.29	4.70	6.02	5.96	0.35	0.12	0.27	0.16
miR-17-5p	8.66	8.36	8.17	10.04	0.85	1.41	1.48	0.79
miR-92a-3p	8.73	8.80	8.34	8.75	0.18	0.90	1.37	1.13
miR-20a-5p	8.84	7.92	8.27	10.18	1.22	0.53	0.80	0.17
miR-23a-3p	8.90	7.88	8.45	7.13	0.45	0.79	0.33	1.63
miR-25-3p	8.94	7.57	7.43	8.11	0.08	0.81	0.92	1.47
miR-15b-5p	9.10	9.07	8.62	9.51	0.56	1.05	1.01	1.51
miR-191-5p	9.36	7.09	8.59	9.12	0.78	0.70	0.81	1.59
miR-30e-5p	9.46	10.24	8.37	9.86	1.15	1.12	0.85	1.17
miR-24-3p	9.70	8.23	7.62	7.80	0.21	0.86	0.94	1.54
miR-106a-5p	9.74	9.04	8.62	9.38	0.87	0.74	0.51	1.55
miR-93-5p	9.76	8.78	7.23	9.42	0.99	1.10	0.66	0.80
miR-223-3p	9.85	8.86	8.75	10.45	1.10	1.11	1.29	1.25
miR-22-3p	9.88	7.73	9.01	9.80	0.11	0.17	0.13	0.29
miR-26b-5p	9.89	11.87	9.78	11.42	0.69	1.34	0.93	1.34
miR-150-5p	9.95	10.15	10.01	10.54	0.74	1.31	0.96	0.85
miR-106b-5p	10.49	8.36	9.66	10.28	0.23	0.20	0.13	0.16
miR-15a-5p	10.76	10.79	11.22	10.37	0.99	0.68	0.84	1.72
miR-19b-3p	10.77	8.28	9.80	10.55	0.19	0.19	0.27	0.11
miR-126a-3p	10.88	10.67	9.04	10.04	0.21	1.17	0.41	1.41
miR-19a-3p	10.90	8.40	9.93	10.42	0.23	0.20	0.27	0.23
miR-128-3p	11.08	10.02	10.34	11.95	1.33	1.65	1.24	1.02
let-7a-5p	11.12	9.92	11.21	11.92	0.92	1.04	1.12	0.65
let-7c-5p	11.36	10.42	10.24	11.31	0.40	0.55	1.28	1.15
miR-18a-5p	11.50	10.14	10.48	11.46	1.22	1.18	0.96	1.57
miR-145a-5p	11.53	11.65	10.65	12.35	1.54	0.69	1.35	0.67
miR-143-3p	11.58	12.62	11.24	11.19	0.83	0.56	0.78	1.39
miR-30d-5p	11.66	10.54	11.22	11.77	0.69	1.14	0.90	1.30
miR-222-3p	11.88	11.25	10.33	11.72	0.64	0.42	0.44	1.13
miR-148a-3p	12.20	11.39	11.09	11.36	0.99	0.22	1.48	1.35
miR-215-5p	12.29	13.33	11.49	13.52	1.00	0.38	0.88	0.64
miR-26a-5p	12.30	10.89	11.61	12.90	0.29	0.67	0.98	1.03
miR-192-5p	12.45	11.58	12.24	12.42	0.60	1.35	0.89	1.01
miR-146a-5p	12.55	13.47	12.27	12.53	0.69	1.17	1.26	1.36

miR-210-3p	12.61	11.99	12.57	13.03	0.51	0.29	0.03	0.95
miR-103-3p	12.75	11.22	12.90	13.74	0.97	0.57	0.63	1.28
miR-203-3p	12.80	12.58	13.90	14.10	0.62	1.11	0.97	0.70
miR-122-5p	13.24	10.90	12.34	10.90	0.30	0.15	0.18	0.25
miR-205-5p	13.63	13.33	14.38	11.84	0.22	0.14	0.26	0.38
miR-125b-5p	13.70	13.60	13.19	12.68	0.21	0.09	0.10	0.13
miR-10b-5p	14.04	14.61	14.17	14.56	0.37	0.33	0.73	1.18
miR-221-3p	14.08	11.78	12.96	13.95	0.18	0.19	0.21	0.19
miR-375-3p	14.38	14.04	12.09	13.53	0.63	0.62	0.55	1.36
miR-181a-5p	14.45	12.30	16.20	16.11	0.45	0.51	0.41	1.13
miR-423-5p	14.61	12.57	13.81	14.31	0.25	0.28	0.15	0.12
miR-134-5p	14.70	12.06	13.72	14.70	0.23	0.30	0.16	0.20
miR-181b-5p	14.78	12.49	13.49	15.75	0.07	0.94	1.00	1.04
miR-130b-3p	14.79	11.62	13.60	14.88	0.26	0.23	0.24	0.27
miR-574-3p	14.93	14.91	14.07	13.82	0.29	0.12	0.27	0.12
miR-214-3p	15.06	13.18	15.43	13.25	0.60	1.26	0.67	0.36
miR-200b-3p	15.14	12.60	15.24	14.30	1.05	0.75	0.39	0.71
miR-34a-5p	15.19	11.92	13.43	13.62	1.27	0.85	0.16	1.04
miR-100-5p	15.20	12.74	14.27	13.59	0.26	1.09	0.90	1.18
miR-10a-5p	15.23	12.59	14.01	12.86	1.69	1.42	0.70	1.71
miR-17-3p	15.29	11.23	14.30	14.72	1.42	0.48	0.66	0.87
miR-155-5p	15.55	12.90	14.21	13.52	0.19	0.26	0.14	0.14
miR-200a-3p	16.07	13.55	15.49	14.39	0.73	0.58	1.58	0.91
miR-296-5p	16.28	10.60	13.59	14.21	1.47	1.16	0.73	1.40
miR-1a-3p	16.29	10.09	16.57	12.97	1.14	0.59	0.69	0.79
miR-200c-3p	16.29	14.09	15.05	15.45	1.07	0.97	1.09	0.97
miR-7a-5p	16.33	13.55	15.24	15.47	1.27	0.97	1.15	1.42
miR-31-5p	16.43	13.36	15.10	16.36	1.23	1.40	0.95	1.10
miR-107-3p	17.30	12.95	16.71	17.00	0.82	0.54	0.45	0.72
miR-141-3p	17.81	13.20	16.21	16.04	0.18	0.91	1.33	0.43
miR-193a-3p	18.08	11.92	16.33	14.24	1.06	1.04	0.94	0.89
miR-224-5p	18.25	15.38	17.71	16.98	0.72	1.37	1.03	0.40
miR-199a-5p	18.32	15.55	18.56	16.62	0.55	1.21	0.35	1.23
miR-124-3p	18.38	16.03	16.02	17.02	0.56	0.56	0.22	1.10
miR-196a-5p	18.48	17.02	19.22	18.04	0.64	0.66	1.53	0.93
miR-376c-3p	18.57	15.31	18.14	14.53	1.25	1.14	0.52	1.49
miR-133a-3p	18.92	13.86	17.72	15.21	0.36	0.59	0.90	1.12
miR-206-3p	19.03	15.00	18.52	16.58	0.91	0.80	1.23	0.94
miR-204-5p	19.28	17.03	18.74	17.66	1.38	0.32	0.67	1.43
miR-211-5p	20.02	17.46	19.31	19.78	0.86	0.94	0.83	0.17
miR-499-5p	20.25	17.57	18.71	18.15	1.15	1.06	0.89	0.77
miR-9-5p	20.35	19.60	19.68	20.23	0.51	0.34	0.13	0.60
miR-34c-5p	20.39	17.52	19.50	19.32	0.08	1.13	0.31	1.47
miR-133b-3p	20.48	14.63	20.06	15.67	0.73	0.47	0.94	1.44
miR-96-5p	ND	ND	ND	ND	ND	ND	ND	ND
miR-184-3p	ND	ND	ND	ND	ND	ND	ND	ND

ND: not detected (Ct > 35).

Table S3 The fold changes in the serum EV microRNAs (pre-normalized to the amount of EVs) in the bleomycin-induced lung fibrosis model mice compared with the non-treated controls.

	Fold Chage			*P* value		
NAME	Day 7	Day 14	Day 28	Day 7	Day 14	Day 28
let-7a-5p	2.30	0.94	0.57	0.225	0.995	0.319
let-7c-5p	1.92	2.17	1.03	0.083	0.200	0.680
miR-1a-3p†	73.49	0.82	9.99	0.014	0.616	0.052
miR-7a-5p†	6.86	2.13	1.81	0.057	0.379	0.408
miR-9-5p†	1.69	1.59	1.09	0.093	0.060	0.766
miR-10a-5p	6.22	2.32	5.17	0.120	0.531	0.151
miR-10b-5p	0.67	0.91	0.70	0.128	0.956	0.600
miR-15a-5p	0.98	0.72	1.31	0.863	0.518	0.448
miR-15b-5p	1.02	1.39	0.75	0.777	0.433	0.994
miR-16-5p	1.08	2.22	0.75	0.722	0.348	0.661
miR-17-3p	16.64	1.98	1.48	0.007	0.525	0.805
miR-17-5p	1.23	1.41	0.38	0.610	0.520	0.168
miR-18a-5p	2.56	2.02	1.02	0.308	0.330	0.892
miR-19a-3p	5.68	1.96	1.40	0.000	0.014	0.074
miR-19b-3p	5.60	1.96	1.16	0.000	0.014	0.150
miR-20a-5p	1.89	1.49	0.39	0.416	0.678	0.241
miR-21a-5p	12.10	4.85	5.03	0.000	0.002	0.000
miR-22-3p	4.44	1.84	1.06	0.000	0.001	0.619
miR-23a-3p	2.03	1.37	3.43	0.168	0.231	0.128
miR-24-3p	2.77	4.21	3.74	0.080	0.088	0.159
miR-25-3p	2.58	2.85	1.78	0.069	0.092	0.230
miR-26a-5p	2.66	1.61	0.66	0.058	0.333	0.583
miR-26b-5p	0.25	1.08	0.35	0.054	0.770	0.108
miR-27a-3p	1.38	0.88	0.70	0.625	0.587	0.406
miR-29a-3p	2.45	1.21	0.36	0.190	0.663	0.189
miR-30d-5p	2.18	1.36	0.93	0.257	0.530	0.858
miR-30e-5p	0.58	2.13	0.76	0.447	0.274	0.721
miR-31-5p†	8.38	2.51	1.05	0.179	0.263	0.995
miR-34a-5p	9.64	3.39	2.96	0.039	0.049	0.224
miR-34c-5p†	7.27	1.85	2.10	0.140	0.023	0.336
miR-92a-3p	0.95	1.31	0.99	0.846	0.487	0.720
miR-93-5p	1.97	5.75	1.27	0.355	0.053	0.764
miR-96-5p	ND	ND	ND	ND	ND	ND
miR-100-5p	5.51	1.91	3.05	0.145	0.168	0.210
miR-103-3p	2.89	0.90	0.50	0.088	0.702	0.424
miR-106a-5p	1.62	2.18	1.28	0.386	0.156	0.542
miR-106b-5p	4.36	1.77	1.16	0.001	0.004	0.256
miR-107-3p†	20.44	1.50	1.23	0.014	0.409	0.714
miR-122-5p	5.08	1.86	5.07	0.000	0.011	0.002
miR-124-3p†	5.10	5.14	2.57	0.030	0.002	0.172

miR-125b-5p	1.07	1.42	2.03	0.499	0.014	0.002
miR-126a-3p	1.16	3.58	1.79	0.540	0.014	0.233
miR-128-3p	2.08	1.68	0.55	0.287	0.451	0.349
miR-130b-3p	9.00	2.28	0.94	0.001	0.006	0.689
miR-133a-3p†	33.29	2.30	13.04	0.017	0.108	0.104
miR-133b-3p†	57.38	1.33	28.04	0.004	0.529	0.066
miR-134-5p	6.23	1.97	1.00	0.003	0.004	0.995
miR-141-3p†	24.46	3.02	3.42	0.050	0.248	0.017
miR-143-3p	0.48	1.26	1.31	0.201	0.662	0.534
miR-145a-5p	0.92	1.84	0.57	0.589	0.404	0.282
miR-146a-5p	0.53	1.21	1.01	0.403	0.594	0.732
miR-148a-3p	1.76	2.16	1.80	0.330	0.383	0.334
miR-150-5p	0.87	0.96	0.66	0.920	0.974	0.438
miR-155-5p	6.29	2.54	4.10	0.001	0.001	0.000
miR-181a-5p†	4.44	0.30	0.32	0.017	0.021	0.072
miR-181b-5p†	4.89	2.45	0.51	0.060	0.150	0.234
miR-184-3p	ND	ND	ND	ND	ND	ND
miR-191-5p	4.83	1.71	1.18	0.046	0.348	0.586
miR-192-5p	1.82	1.15	1.02	0.309	0.664	0.800
miR-193a-3p†	71.46	3.36	14.29	0.086	0.148	0.030
miR-195a-5p	2.41	1.10	0.79	0.202	0.922	0.714
miR-196a-5p†	2.75	0.60	1.36	0.073	0.787	0.420
miR-199a-5p	6.82	0.85	3.27	0.064	0.506	0.111
miR-200a-3p	5.71	1.49	3.20	0.030	0.471	0.134
miR-200b-3p	5.79	0.93	1.79	0.039	0.665	0.355
miR-200c-3p†	4.60	2.36	1.78	0.075	0.241	0.296
miR-203-3p	1.16	0.47	0.41	0.608	0.213	0.100
miR-204-5p†	4.77	1.46	3.07	0.014	0.713	0.175
miR-205-5p	1.23	0.59	3.45	0.106	0.018	0.012
miR-206-3p†	16.35	1.42	5.47	0.055	0.548	0.064
miR-210-3p	1.53	1.03	0.75	0.153	0.935	0.698
miR-211-5p†	5.91	1.64	1.19	0.059	0.385	0.850
miR-214-3p†	3.67	0.77	3.52	0.109	0.500	0.010
miR-215-5p	0.48	1.73	0.42	0.180	0.363	0.162
miR-221-3p	4.92	2.18	1.09	0.000	0.003	0.450
miR-222-3p	1.54	2.92	1.11	0.217	0.033	0.655
miR-223-3p	1.99	2.15	0.66	0.403	0.309	0.619
miR-224-5p†	7.33	1.45	2.42	0.160	0.464	0.038
miR-296-5p†	51.28	6.44	4.21	0.055	0.068	0.173
miR-375-3p	1.26	4.90	1.80	0.529	0.016	0.370
miR-376c-3p†	9.56	1.35	16.42	0.099	0.902	0.097
miR-423-5p	4.11	1.74	1.23	0.003	0.007	0.123
miR-499-5p†	6.40	2.90	4.30	0.121	0.216	0.068
miR-574-3p	1.01	1.81	2.16	0.987	0.026	0.002

ND: not detected (Ct > 35).

[†]: microRNAs for which the Ct in either group was > 30, which indicated that the expression levels were relatively low and could cause greater variations in the fold-change results.

Table S4 The fold-changes of serum EV microRNAs (post-normalized to the amount of EVs) in the bleomycin-induced lung fibrosis model mice compared with the non-treated controls.

NAME	Day 7		Day 14		Day 28	
	Fold change	q value	Fold change	q value	Fold change	q value
let-7a-5p	1.70	0.205	1.20	0.466	0.82	0.828
let-7c-5p	1.43	0.292	2.78	0.956	1.47	0.543
miR-1a-3p†	54.44*	0.048	1.05	0.745	14.28	0.325
miR-7a-5p†	5.08	0.155	2.73	0.651	2.59	0.659
miR-9-5p†	1.25	0.193	2.04	0.333	1.55	0.877
miR-10a-5p	4.61	0.214	2.97	0.709	7.38	0.454
miR-10b-5p	0.50	0.211	1.17	0.993	1.00	0.783
miR-15a-5p	0.73	0.842	0.93	0.704	1.87	0.948
miR-15b-5p	0.76	0.848	1.78	0.708	1.07	0.736
miR-16-5p	0.80	0.851	2.84	0.616	1.06	0.830
miR-17-3p	12.33*	0.022	2.54	0.705	2.12	0.878
miR-17-5p	0.91	0.724	1.81	0.713	0.55	0.465
miR-18a-5p	1.90	0.375	2.59	0.622	1.46	0.920
miR-19a-3p	4.10*	0.008	2.51	0.059	1.99	0.199
miR-19b-3p	4.07*	0.009	2.51	0.051	1.66	0.385
miR-20a-5p	1.40	0.508	1.91	0.804	0.56	0.498
miR-21-5p	9.29*	0.003	5.77*	0.036	7.97*	0.001
miR-22-3p	3.20*	0.012	2.35	0.055	1.52	0.797

miR-23a-3p	1.51	0.224	1.75	0.382	4.90	0.418
miR-24-3p	2.05	0.160	5.40	0.240	5.34	0.450
miR-25-3p	1.91	0.164	3.65	0.323	2.54	0.492
miR-26a-5p	1.97	0.154	2.07	0.836	0.94	0.390
miR-26b-5p	0.19	0.182	1.38	0.625	0.49	0.808
miR-27a-3p	1.02	0.719	1.12	0.690	1.00	0.628
miR-29a-3p	1.81	0.333	1.55	0.805	0.52	0.451
miR-30d-5p	1.61	0.359	1.74	0.701	1.33	0.922
miR-30e-5p	0.43	0.550	2.73	0.631	1.09	0.835
miR-31-5p[†]	6.21	0.259	3.22	0.620	1.50	0.957
miR-34a-5p	7.14	0.160	4.35	0.168	4.23	0.444
miR-34c-5p[†]	5.39	0.220	2.37	0.205	2.99	0.591
miR-92a-3p	0.71	0.918	1.68	0.701	1.41	0.855
miR-93-5p	1.46	0.446	7.37	0.204	1.81	0.867
miR-96-5p	ND		ND		ND	
miR-100-5p	4.08	0.220	2.45	0.385	4.36	0.449
miR-103-3p	2.14	0.211	1.16	0.815	0.72	0.664
miR-106a-5p	1.20	0.483	2.80[*]	0.049	1.83	0.464
miR-106b-5p	3.51[*]	0.004	2.27	0.431	1.65	0.793
miR-107-3p[†]	15.14[*]	0.041	1.93	0.610	1.76	0.831
miR-122-5p	4.07[*]	0.004	2.39	0.052	7.24[*]	0.006
miR-124-3p[†]	3.78	0.157	6.60	0.052	3.67	0.437
miR-125b-5p	0.80	0.710	1.83	0.089	3.40[*]	0.010

miR-126a-3p	0.86	0.644	4.59	0.085	2.56	0.487
miR-128-3p	1.54	0.379	2.15	0.671	0.78	0.562
miR-130b-3p	7.50*	0.006	3.00	0.055	1.34	0.825
miR-133a-3p†	24.66*	0.016	2.94	0.708	18.62	0.349
miR-133b-3p†	42.50	0.050	1.71	0.381	40.06	0.403
miR-134-5p	4.81*	0.008	2.53	0.080	1.43	0.983
miR-141-3p†	18.12	0.147	3.88	0.510	4.88	0.170
miR-143-3p	0.36	0.259	1.62	0.770	1.87	0.768
miR-145a-5p	0.68	0.715	2.35	0.647	0.81	0.554
miR-146a-5p	0.39	0.445	1.55	0.725	1.45	0.856
miR-148a-3p	1.31	0.379	2.77	0.650	2.57	0.562
miR-150-5p	0.65	0.979	1.23	0.986	0.95	0.693
miR-155-5p	4.28*	0.009	3.26*	0.039	6.37*	0.001
miR-181a-5p†	3.29	0.097	0.38	0.164	0.45	0.377
miR-181b-5p†	3.62	0.157	3.14	0.380	0.73	0.445
miR-184-3p	ND		ND		ND	
miR-191-5p	3.58	0.158	2.20	0.609	1.69	0.814
miR-192-5p	1.35	0.416	1.48	0.773	1.46	0.873
miR-193a-3p†	52.93	0.188	4.31	0.461	20.42	0.206
miR-195a-5p	1.79	0.314	1.40	0.976	1.13	0.828
miR-196a-5p†	2.04	0.152	0.77	0.794	1.94	0.723
miR-199a-5p†	5.05	0.171	1.09	0.710	4.67	0.384
miR-200a-3p	4.23	0.085	1.91	0.717	4.58	0.388

miR-200b-3p	4.29	0.159	1.20	0.515	2.55	0.546
miR-200c-3p†	3.41	0.112	3.03	0.797	2.55	0.554
miR-203-3p	0.86	0.710	0.60	0.501	0.58	0.414
miR-204-5p†	3.53	0.077	1.87	0.829	4.38	0.459
miR-205-5p	0.91	0.167	0.76	0.078	5.16	0.063
miR-206-3p†	12.11	0.176	1.82	0.702	7.82	0.340
miR-210-3p	1.14	0.256	1.31	0.968	1.07	0.820
miR-211-5p†	4.38	0.172	2.10	0.656	1.70	0.878
miR-214-3p†	2.72	0.205	0.99	0.697	5.02	0.066
miR-215-5p	0.36	0.273	2.22	0.598	0.61	0.451
miR-221-3p	3.64*	0.004	2.79	0.053	1.56	0.745
miR-222-3p	1.14	0.321	3.74	0.088	1.59	0.822
miR-223-3p	1.47	0.519	2.76	0.620	0.94	0.806
miR-224-5p†	5.43	0.236	1.86	0.696	3.45	0.276
miR-296-5p†	37.98	0.157	8.26	0.242	6.01	0.451
miR-375-3p	0.94	0.589	6.28	0.115	2.58	0.629
miR-376c-3p†	7.08	0.223	1.73	0.998	23.46	0.380
miR-423-5p	3.09*	0.011	2.22	0.062	1.76	0.410
miR-499-5p†	4.74	0.220	3.72	0.491	6.14	0.395
miR-574-3p	0.75	0.991	2.33	0.062	3.53*	0.006

*q *value* < 0.05 compared with non-treated control.

ND: not detected (Ct > 35).

†: microRNAs for which the Ct in either group was > 30, which indicated that the expression

levels were relatively low and could cause greater variations in the fold-change results.

Table S5 The delta Ct values (miR-23a-3p - miR-451a) for the mouse serum EV samples used for the MicroRNA PCR array.

sample	Sample Ct		delta Ct(miR-23a-3p - miR-451a)*
	miR-23a-3p	miR-451a	
Control #1	22.97	18.85	4.13
Control #2	23.13	18.37	4.76
Control #3	23.37	18.86	4.51
BLM-7d #1	21.61	17.37	4.24
BLM-7d #2	21.74	17.67	4.07
BLM-7d #3	22.15	18.00	4.15
BLM-14d #1	22.66	18.28	4.38
BLM-14d #2	22.67	17.94	4.74
BLM-14d #3	22.32	17.57	4.76
BLM-28d #1	23.38	18.97	4.41
BLM-28d #2	23.44	18.99	4.46
BLM-28d #3	22.10	17.69	4.41

Table S6 The baseline characteristics of the COPD patients.

	COPD (n=24)
Sex, male, n (%)	20 (83)
Age, median (IQR)	71 (68-79)
% predicted FVC, median (IQR)	89 (76-105)
% predicted FEV_1, median (IQR)	56 (36-79)
FEV1/FVC (%), median (IQR)	46 (30-52)
Smoking history, n (%)	24 (100)

The data are expressed as the median values (IQR) or n (%). FVC: forced vital capacity; FEV_1: forced expiratory volume in one second.

Table S7 Differential blood cell counts of the two groups into which IPF patients were divided on the basis of the cut-off value of the adjusted levels of serum EV miR-21-5p.

	miR-21-5p (copies/SI)		
	< 2.1 (n = 20)	≥2.1 (n = 21)	*P* value
White blood cells (/μL)	5300 (5600-8450)	7100 (5800-7500)	0.70
Red Blood cells ($x10^6$/μL)	428 (397-465)	414 (368-430)	0.12
Platelets ($x10^4$/μL)	24.7 (18.7-29.2)	22.3 (17.6-35.1)	0.79
Neutrophils (/μL)	4030 (3080-5770)	3960 (3450-4830)	0.90
Lymphocytes (/μL)	1880 (1440-2380)	1780 (1010-2320)	0.36
Monocytes (/μL)	380 (310-440)	410 (340-490)	0.43
Eosinophils (/μL)	140 (80 - 250)	200 (110-300)	0.29
Basophils (/μL)	20 (10-40)	30 (20-50)	0.11

The data are expressed as the medians (IQR). The differences between the two groups were analysed using the Mann-Whitney U test.

Table S8 The relationship between the non-normalized levels of EV miR-21-5p and the clinical variables in the 41 IPF patients.

	miR-21-5p (copies/mL)	
	r_s	*P*
% predicted VC	- 0.28	0.07
KL-6	0.21	0.19
SP-D	0.37	**0.02**
LDH	0.19	0.23
rate of decline in % predicted VC over 6 months	0.41	**0.01**

r_S: Spearman rank correlation coefficient. The correlations were calculated using the Spearman rank correlation.

Bold denotes $p < 0.05$. VC: vital capacity; KL-6: Krebs von den Lungen-6; SP-D: surfactant protein D.

Table S9 Univariate and multivariate Cox hazards model analyses for mortality during the 30-month follow-up period in IPF patients.

	Univariate		Multivariate	
	HR (95% CI)	*P* value	HR (95% CI)	*P* value
Sex				
Male	0.60 (0.17-2.78)	0.48		
Female	1.64 (0.35-5.75)	0.48		
Age	1.09 (1.001-1.20)†	**0.04**	1.04 (0.95-1.16)	0.31
% predicted VC	0.98 (0.95-1.01)†	0.31		
Emphysematous lesion detected by CT	2.20 (0.33-8.06)	0.35		
Rate (%) of decline in VC per 6 months	1.04 (0.98-1.09)†	0.11		
KL-6	0.99 (0.99-1.00)†	0.85		
SP-D	1.00 (0.99-1.00)†	0.41		
LDH	0.99 (0.97-1.00)†	0.23		
miR-21-5p (copies/mL)	1.44 (1.10-1.83)	**0.009**	1.33 (1.0007-1.76)	**0.04**

Bold represents $p < 0.05$. HR: hazard ratio; CI: confidence interval. VC: vital capacity; KL-6: Krebs von den Lungen-6; SP-D: surfactant protein D

†: A unit hazard ratio (hazard ratio per one unit change in each regressor) is shown.

Table S10 Characteristics of the two groups into which IPF patients were divided on the basis of the median of the non-normalized levels of serum EV miR-21-5p.

	miR-21-5p ($x10^7$ copies/mL)		
	< 1.25	≥ 1.25	*P* value
	(n = 20)	(n = 21)	
Age, yr	71 (65-76)	75 (70-82)	**0.04**
Sex, male, n (%)	17 (85)	15 (71)	0.45
Smoking history			
yes, n (%)	19 (95)	15 (71)	0.09
Baseline VC (%)	78 (69-104)	81 (62-91)	0.48
Emphysematous lesion detected by CT, n(%)	3(15)	1(5)	0.28
KL-6	645 (540 - 982)	1040 (706-1921)	**0.04**
SP-D	211 (141-277)	202 (140-320)	0.77
LDH	207 (183-215)	227 (177-280)	0.18

The data are expressed as n (%) or medians (IQR). The differences in age, % VC, or other serum markers between the two groups were analysed using the Mann-Whitney U test. The comparisons of gender, smoking history and emphysema between the two groups were analysed using the Fisher exact test.

Bold represents $p < 0.05$. VC: vital capacity; KL-6: Krebs von den Lungen-6; SP-D: surfactant protein D.

Fig. S1

a

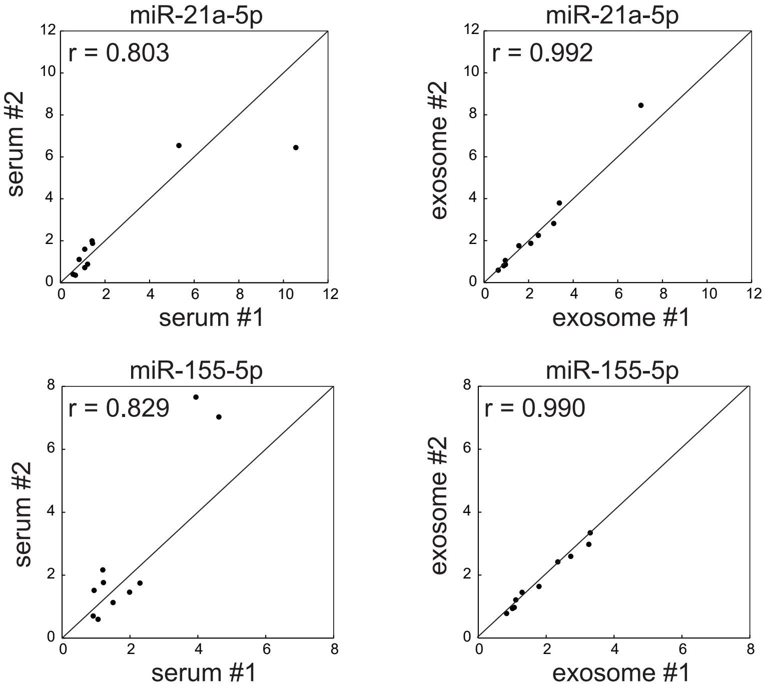

b

miR-21	serum #1	serum #2	exosome #1	exosome #2
serum #1	1.000	0.803	0.768	0.765
serum #2	-	1.000	0.667	0.629
exosome #1	-	-	1.000	0.992
exosome #2	-	-	-	1.000

miR-155	serum #1	serum #2	exosome #1	exosome #2
serum #1	1.000	0.829	0.729	0.719
serum #2	-	1.000	0.767	0.732
exosome #1	-	-	1.000	0.990
exosome #2	-	-	-	1.000

Fig. S2

a

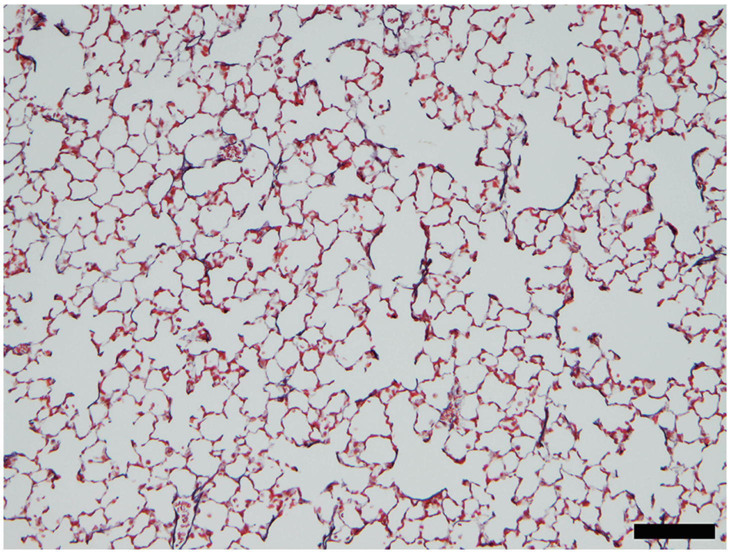

b

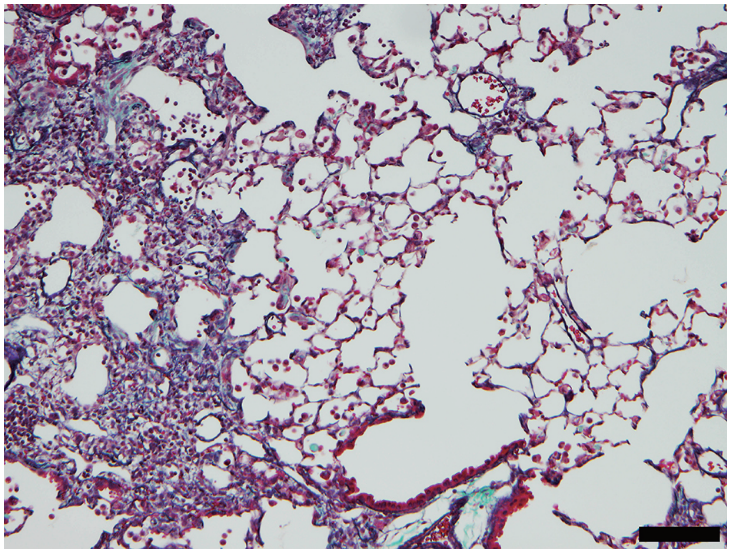

c

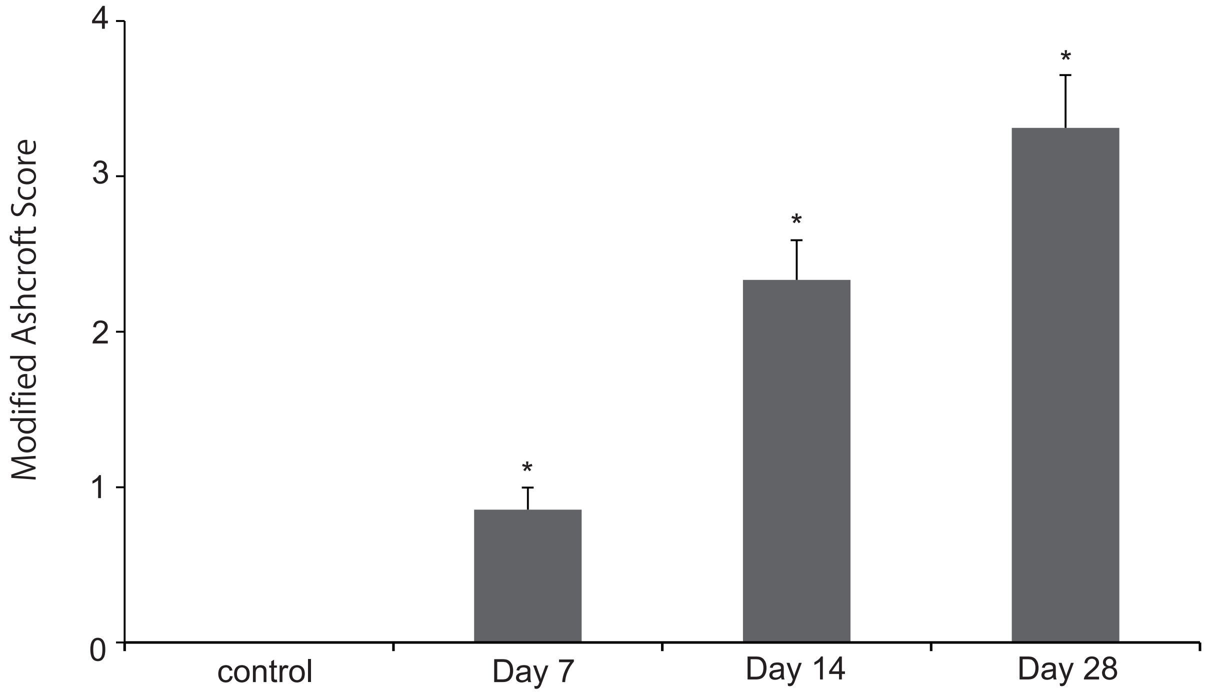

Fig. S3

a

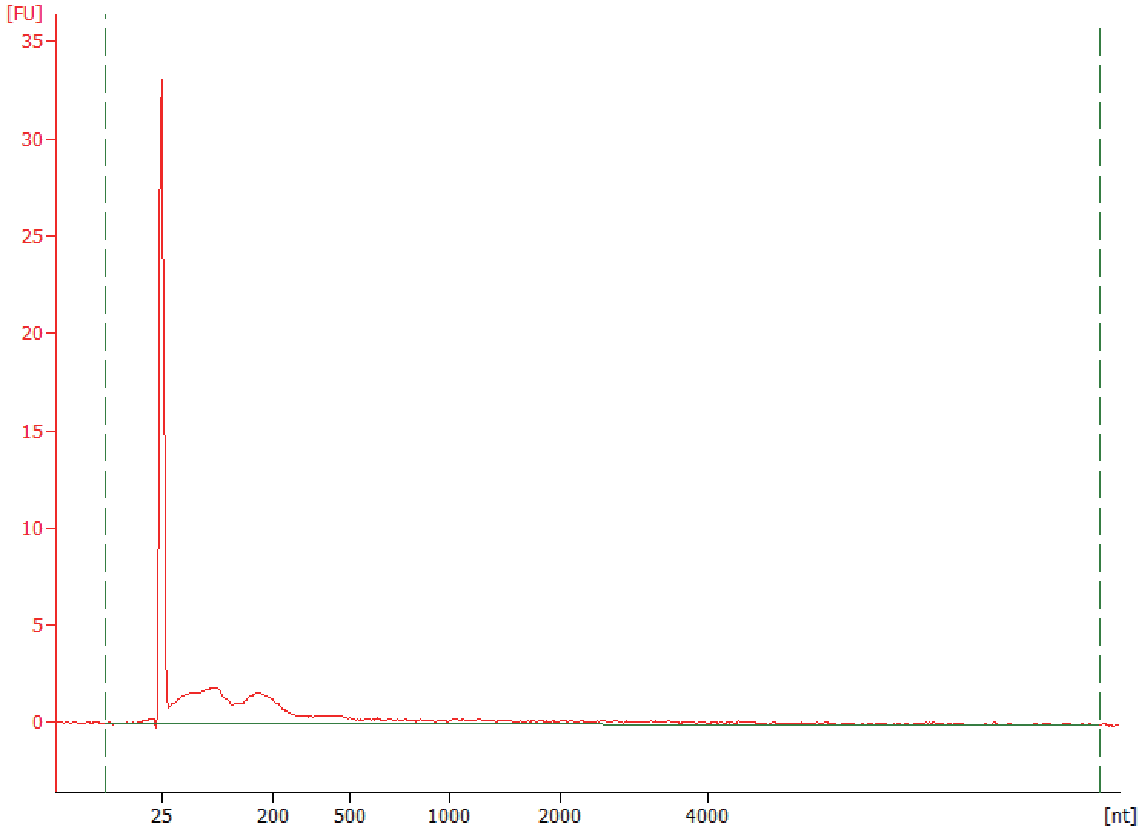
[FU]
35
30
25
20
15
10
5
0
25
200
500
1000
2000
4000
[nt]

b

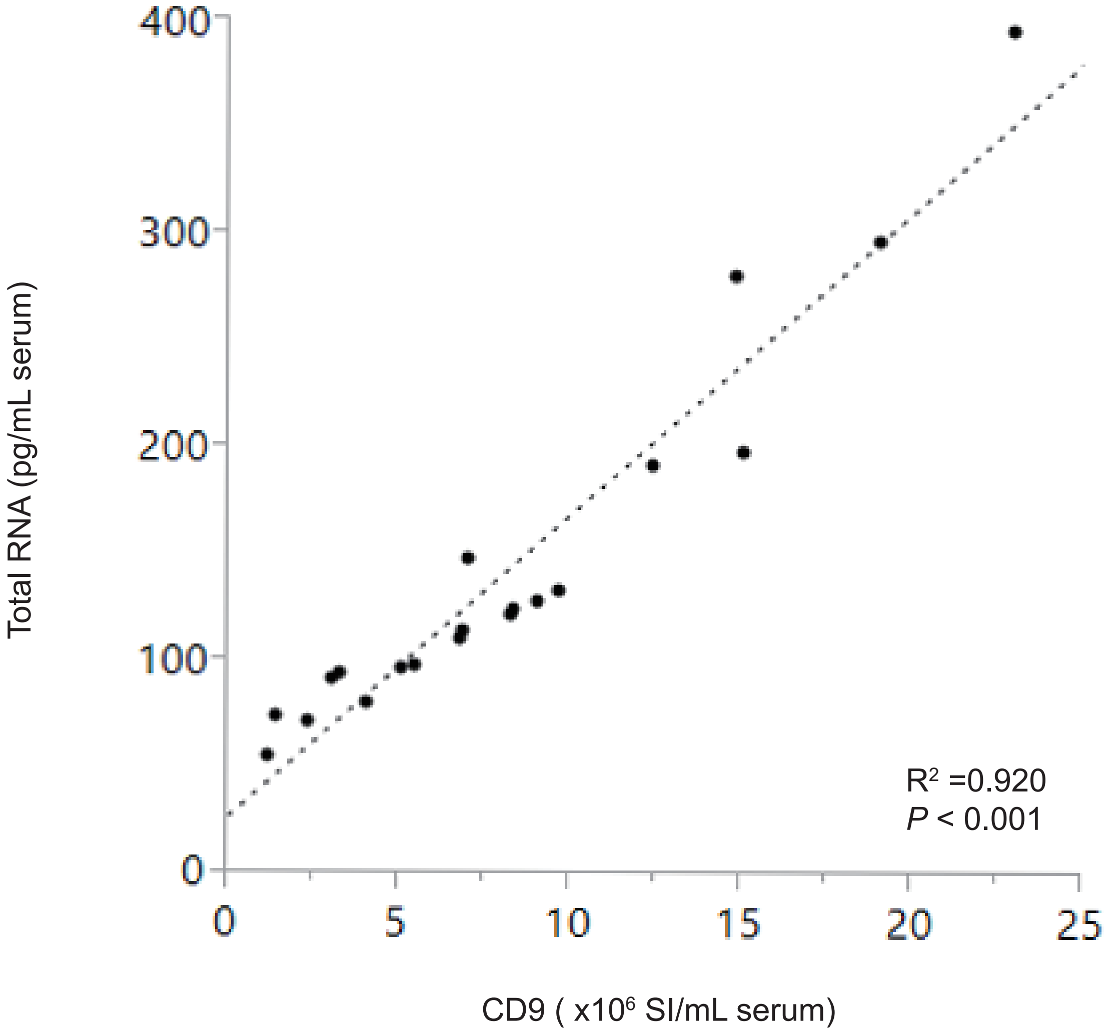
400
300
200
100
0
Total RNA (pg/mL serum)
0
5
10
15
20
25
CD9 (x10⁶ SI/mL serum)
R² =0.920
P < 0.001

Fig. S4

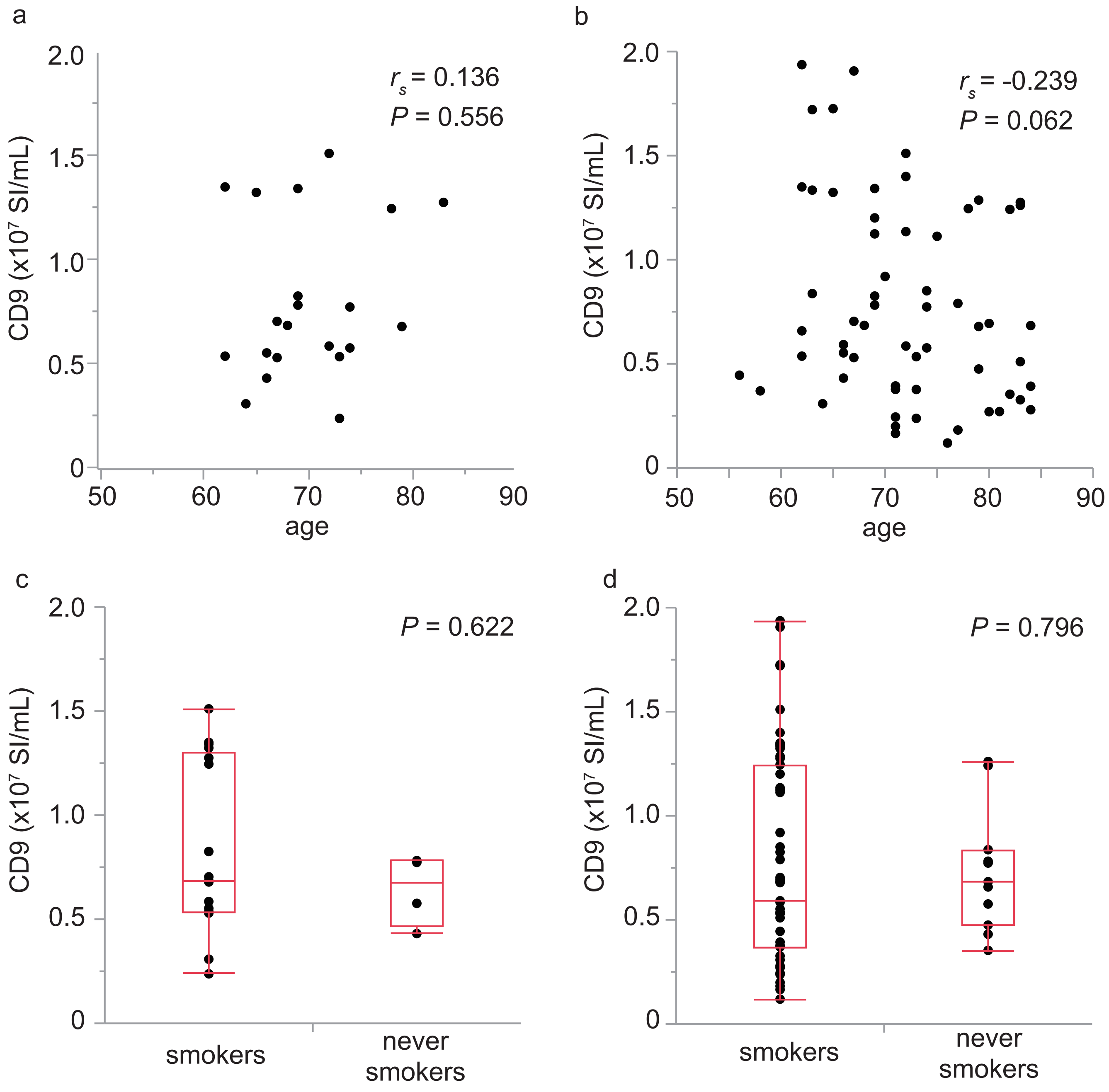
a
b
c
d
CD9 (x10^7 SI/mL)
age
r_s = 0.136
P = 0.556
r_s = -0.239
P = 0.062
P = 0.622
P = 0.796
smokers
never smokers
0
0.5
1.0
1.5
2.0
50
60
70
80
90

Fig. S5

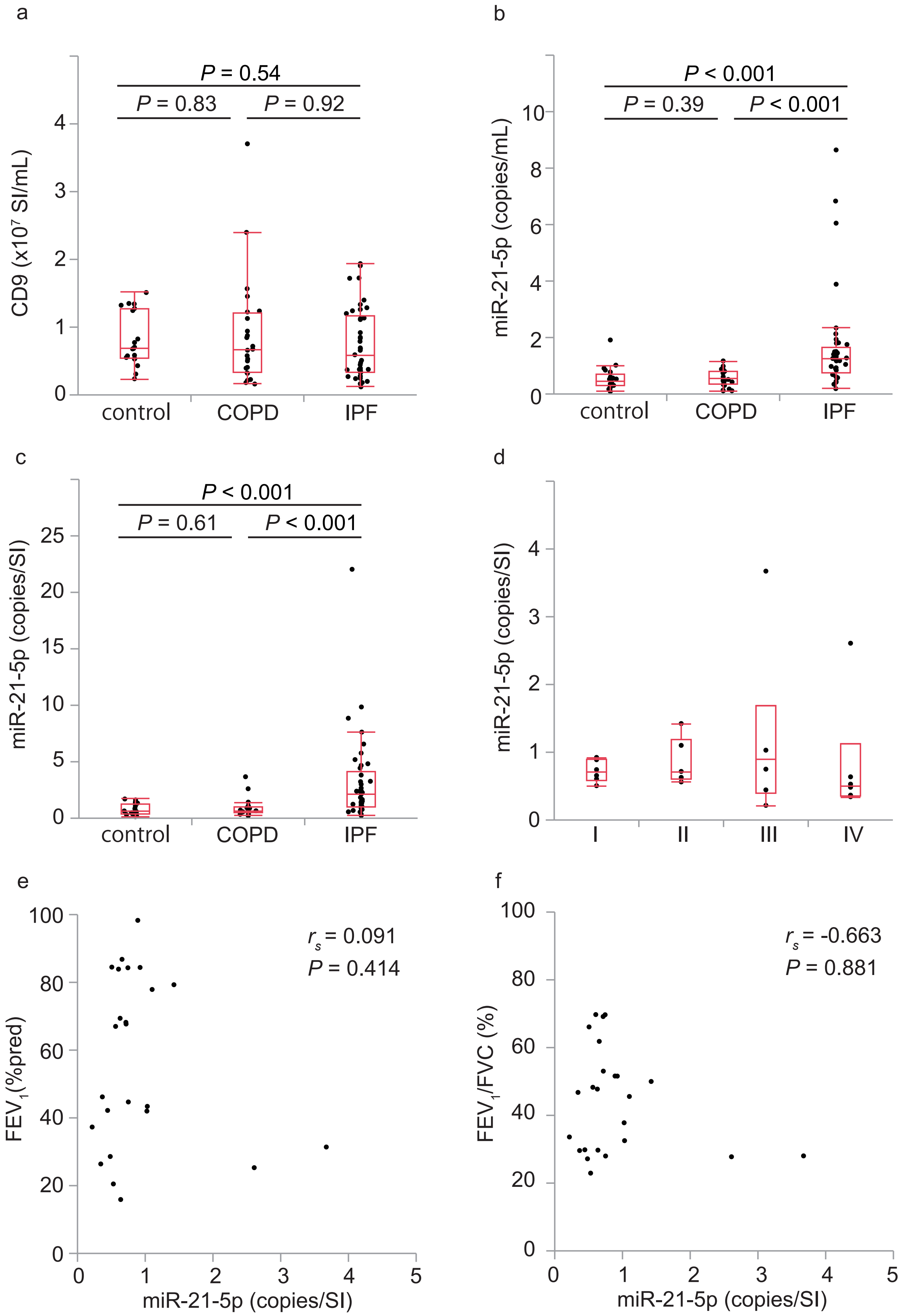
a
P = 0.54
P = 0.83
P = 0.92
CD9 (x10⁷ SI/mL)
0
1
2
3
4
control
COPD
IPF
b
P < 0.001
P = 0.39
P < 0.001
miR-21-5p (copies/mL)
0
2
4
6
8
10
control
COPD
IPF
c
P < 0.001
P = 0.61
P < 0.001
miR-21-5p (copies/SI)
0
5
10
15
20
25
control
COPD
IPF
d
miR-21-5p (copies/SI)
0
1
2
3
4
I
II
III
IV
e
r_s = 0.091
P = 0.414
FEV1(%pred)
0
20
40
60
80
100
0
1
2
3
4
5
miR-21-5p (copies/SI)
f
r_s = -0.663
P = 0.881
FEV1/FVC (%)
0
20
40
60
80
100
0
1
2
3
4
5
miR-21-5p (copies/SI)

Fig. S6

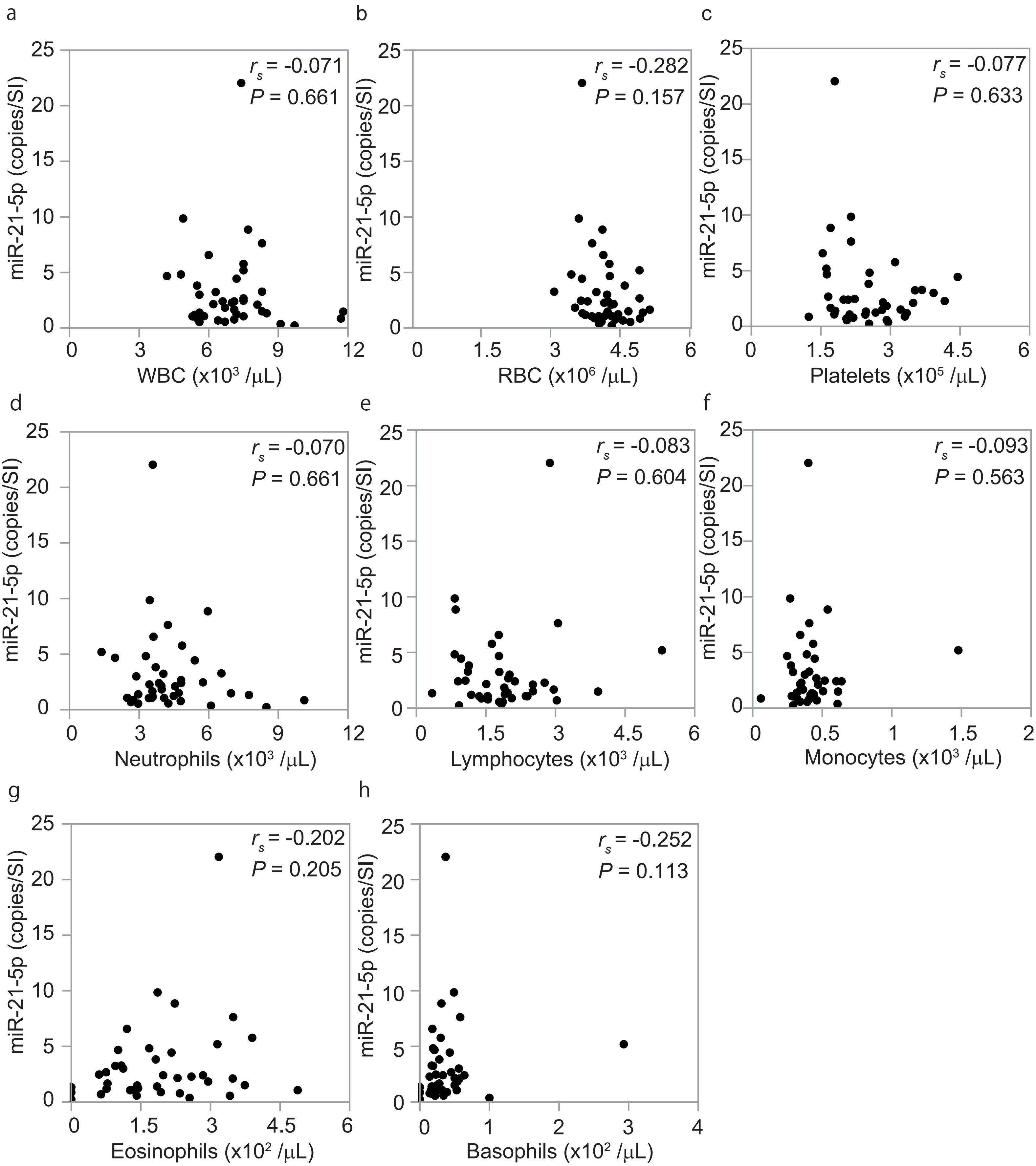
a
b
c
miR-21-5p (copies/SI)
r_s = -0.071
P = 0.661
WBC (x10³ /µL)
r_s = -0.282
P = 0.157
RBC (x10⁶ /µL)
r_s = -0.077
P = 0.633
Platelets (x10⁵ /µL)
d
e
f
r_s = -0.070
P = 0.661
Neutrophils (x10³ /µL)
r_s = -0.083
P = 0.604
Lymphocytes (x10³ /µL)
r_s = -0.093
P = 0.563
Monocytes (x10³ /µL)
g
h
r_s = -0.202
P = 0.205
Eosinophils (x10² /µL)
r_s = -0.252
P = 0.113
Basophils (x10² /µL)

Fig. S7

a

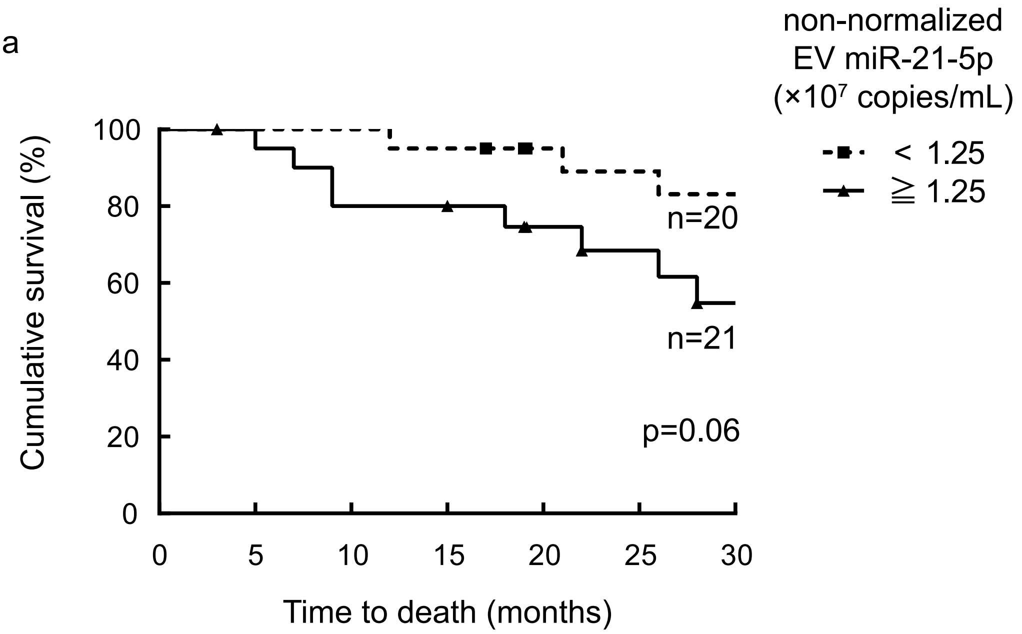
non-normalized
EV miR-21-5p
(×10^7 copies/mL)
< 1.25
≧ 1.25
Cumulative survival (%)
100
80
60
40
20
0
n=20
n=21
p=0.06
0
5
10
15
20
25
30
Time to death (months)

b

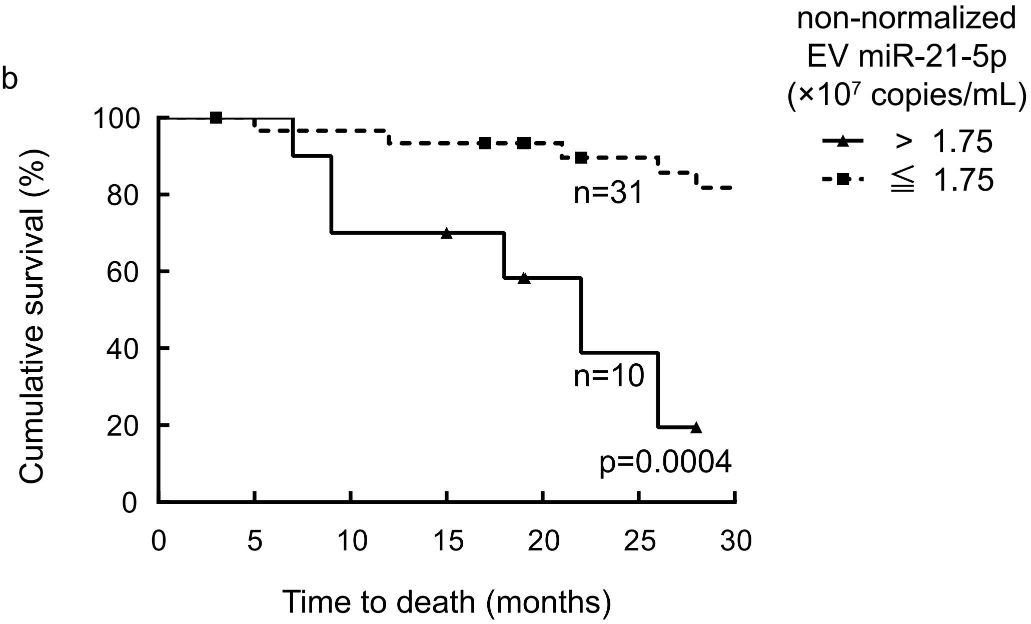
non-normalized
EV miR-21-5p
(×10^7 copies/mL)
> 1.75
≦ 1.75
Cumulative survival (%)
100
80
60
40
20
0
n=31
n=10
p=0.0004
0
5
10
15
20
25
30
Time to death (months)

Fig. S8

a

Pt ID	miR-21-5p at registration (copies/SI)	Rate of decline in %VC over 6M (%)	Dead or alive
IPF 10	2.39	12.23	Dead at 12 months
IPF 11	2.14	0.75	alive
IPF 15	1.05	1.23	alive
IPF 40	4.67	22.93	alive

b

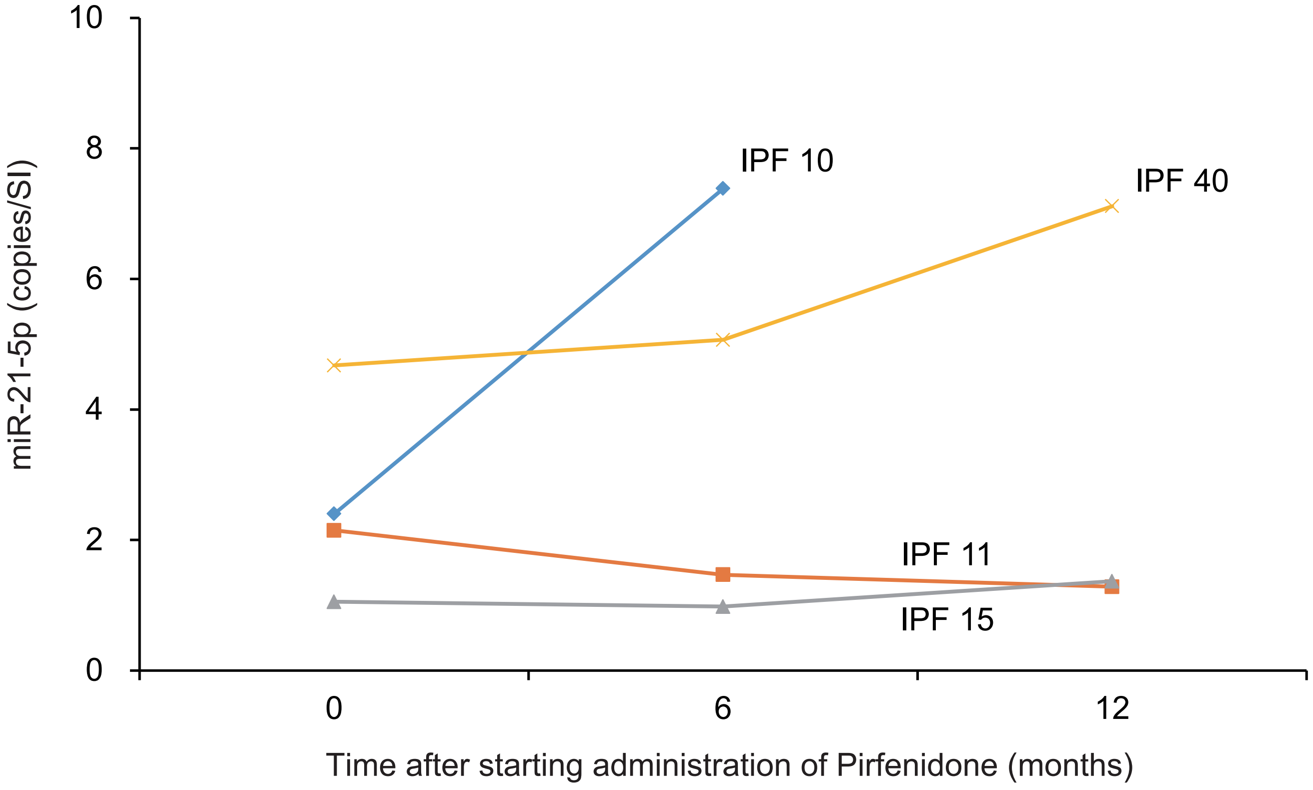

Supplement: Additional file 1: — Online supplemental material. (PDF 9 mb) [file 12931_2016_427_MOESM1_ESM.pdf]
